# Superlattices assembled through shape-induced directional binding

**DOI:** 10.1038/ncomms7912

**Published:** 2015-04-23

**Authors:** Fang Lu, Kevin G. Yager, Yugang Zhang, Huolin Xin, Oleg Gang

**Affiliations:** 1 Center for Functional Nanomaterials, Brookhaven National Laboratory, Upton, New York 11973, USA

## Abstract

Organization of spherical particles into lattices is typically driven by packing considerations. Although the addition of directional binding can significantly broaden structural diversity, nanoscale implementation remains challenging. Here we investigate the assembly of clusters and lattices in which anisotropic polyhedral blocks coordinate isotropic spherical nanoparticles via shape-induced directional interactions facilitated by DNA recognition. We show that these polyhedral blocks—cubes and octahedrons—when mixed with spheres, promote the assembly of clusters with architecture determined by polyhedron symmetry. Moreover, three-dimensional binary superlattices are formed when DNA shells accommodate the shape disparity between nanoparticle interfaces. The crystallographic symmetry of assembled lattices is determined by the spatial symmetry of the block's facets, while structural order depends on DNA-tuned interactions and particle size ratio. The presented lattice assembly strategy, exploiting shape for defining the global structure and DNA-mediation locally, opens novel possibilities for by-design fabrication of binary lattices.

## Abstract (web summary)

Controlling self-assembly of nanoparticles into superlattices is an important approach to build functional materials. Here, Lu *et al*. use directional binding provided by DNA-encoded polyhedral blocks—cubes or octahedrons—to guide spherical nanoparticles into clusters and three-dimensional lattices.

## Graphical abstract

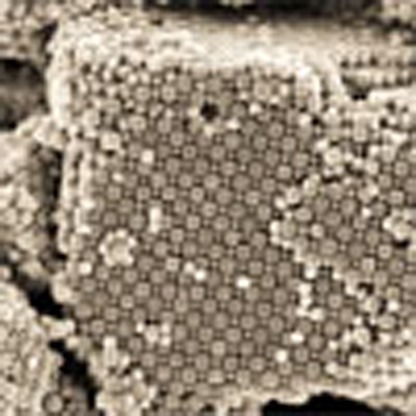

Controlling the organization of nanoparticles in superlattices is important for creating materials with emergent and collective properties for applications in photonics, catalysis, biomaterials and high-energy composites^1^,^2^,^3^. Spherical particles generally assemble into lattice symmetries determined by simple packing criteria. Richer phase behaviour has been observed by exploiting more sophisticated interaction potentials; for instance, exploiting ligand and DNA shells^3^,^4^,^5^,^6^,^7^,^8^, van der Waals^9^, electrostatic^10^,^11^, dipolar^12^,^13^ and magnetic forces^11^. Although much diversity has been demonstrated, the highly system-dependent results point towards difficulties for the predictable fabrication of designed materials.

An alternative paradigm is to introduce directional bonds between spherical particles, thereby coordinating species in the particle's local environment based on the bonding geometry rather than packing. One possible realization is ‘patchy particles'^12^,^14^,^15^,^16^, where spherical particles are decorated with specifically arranged binding regions^17^. The resultant anisotropic interactions mimic the directional bonds of atomic systems. This strategy has been highly successful at the micron scale, with demonstration of assembling designed clusters^17^,^18^. However, as shown by theoretical studies^14^,^19^,^20^, the formation of ordered extended lattices from such particles might be hindered by local orientational defects that cannot easily heal; experimentally, only one-dimensional (1D) and two-dimensional (2D) organizations of colloids were shown^13^,^18^. Recent progress^21^,^22^,^23^ in transferring this concept to the assembly of nano-scale systems is promising; however, it still remains comparatively elusive because of the difficulties in large-scale synthesis of nanoparticles with well-defined anisotropic patches. In this work, we present an alternative idea: the ordering of spherical nanoparticles can be dictated through directional bonds provided by DNA-encoded faceted nanoscale ‘blocks'. We demonstrate, using cubic and octahedral nanoblocks, that well-defined three-dimensional (3D) lattices of spheres and blocks can be formed within appropriate regimes of nanoparticle sizes and DNA shells.

## Results

### Lattices of hetero-shaped particles via directional bonds

**Figure 1:**
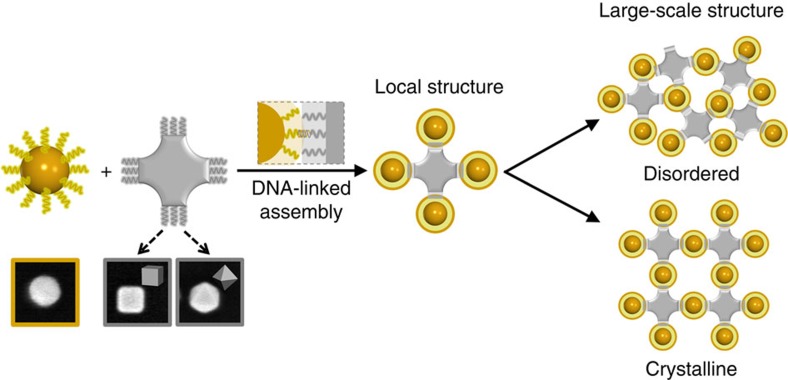
Schematic of shape-induced directional bonds for DNA-linked nanoparticle assembly. SNPs (yellow unit) are isotropic objects with high symmetry. ANPs (grey unit) have low-order symmetry; for example, CB or OC as shown in electron micrographs (inner frame length of images is 100 nm). Regulated interactions between particles are induced by functionalizing SNPs and ANPs with complementary DNA strands. The directional bonding of ANPs coordinates SNPs in a local structure (clusters). Our study examines the conditions under which the shape of ANPs can be translated into well-established local structure, and factors affecting the formation of either disordered or ordered large-scale 3D assembly.

In our approach, schematically shown in Fig. 1, bonding directionality is inherently determined by the block's anisotropic shape through attractive facets. As a result, spheres can be coordinated into clusters in accordance with the geometry of the shaped object. Ultimately, large-scale binary lattices can be formed in a predictable manner. In this study we realize this concept by employing directional bonds provided through polyhedral nano blocks, either cubes (CBs) or octahedrons (OCs), for assembly of spherical nanoparticles. The mutual attraction of heterogeneously shaped particles is required to suppress phase separation^24^,^25^,^26^. In this regard, we functionalize both shaped particles (spherical and polyhedral) with DNA strands, allowing us to ‘encode' particle interactions via DNA sequence. We enforce attractive interactions between different-shape particles via DNA complementary, while same-shape particles are repulsive because of entropic chain effects. In addition, the softness of DNA shell plays an important role in accommodating dissimilar particle curvatures. We stress that in the proposed approach, no complex engineering of spherical particles is required, and this method can take advantage of the wide variety of available nanoscale-shaped objects^27^.

Despite the conceptual simplicity of this approach for generating designed clusters, it is nontrivial to predict the requirements for the formation of extended, ordered 3D superlattices (Fig. 1). Indeed, the geometrical constraints imposed by shapes^26^,^28^,^29^ result in a rich structural diversity even in monocomponent assemblies of shaped objects^30^,^31^,^32^,^33^,^34^,^35^,^36^. Thus, it is not yet clear what dictates or inhibits the formation of large-scale ordered arrays even for the simplest case of hetero-shapes: mutually attractive spherical and shaped objects. At a general level, different particle shapes will exhibit significantly different anisotropic interactions in a system, thereby imposing conflicting local constraints that may override intended bonding directionality. Such conflicts can be resolved via complementary-shaped particles due to the favourable packing^37^,^38^,^39^, certain shapes can be accommodated at the right size regimes^40^ or by optimizing a delicate balance of interactions and space-filling requirements^11^,^41^. In our approach, the use of DNA permits tuning the interaction ‘softness' due to DNA's polymeric nature. This allows us to probe the complex interplay between shape-induced directional binding, the interaction potential and geometrical requirements due to the particle size-ratios. As we show below, ordered assemblies of disparate shapes do not arise generically, even in the presence of well-defined directional bonds. Instead, crystals are obtained only with appropriate selection of interaction potentials and particle size ratio, in particular, those that satisfy local geometric constraints. Thus, directional bonds can be optimized to yield highly ordered binary 3D superlattices of hetero-shaped nanoparticles, wherein global crystallographic symmetry is directly selected by particle shape.

To explore the proposed strategy (Fig. 1) for assembly of a binary assembly of hetero-shaped particles, we used spherical nanoparticles (SNPs) as isotropic objects with highest-order symmetry and anisotropic nanoparticle (ANP), shown as a generic grey block in the schematic. Specifically, polyhedral nano objects, either a cube (CB) or an octahedron (OC), were employed in this study. These two shapes are mathematically dual to one another: the point symmetry of CB facets matches that of OC vertices. By examining these two closely related shapes, we are able to examine the effects of both particle symmetry and geometry on lattice formation. Regulated interactions between particles are induced by functionalizing ANPs and SNPs with complementary DNA strands. We hypothesize that the ability of ANPs to coordinate SNPs is determined by the faces of ANPs, since DNA hybridization is maximized when area between particles with complementary DNA is increased. For example, due to their six square and eight equilateral triangular faces, respectively, the CB and OC are expected to locally coordinate SNPs into six- and eightfold symmetries. On other hand, it is not clear under what conditions disordered versus ordered structures will form for large-scale SNP/ANP assemblies, even if local coordination is precisely controlled. We conjecture that the shape symmetry of ANPs can be translated under certain conditions into the global lattice type; thus, the symmetry of the lattice is encoded by geometrical properties of the ANP, while DNA is responsible for local connections of hetero-shaped objects. The formed lattice can be seen as a result of the constraining of a high-order-symmetry element, such as sphere, by a low-order-symmetry anisotropic object.

### Assembly of cube and sphere nanoparticles

**Figure 2:**
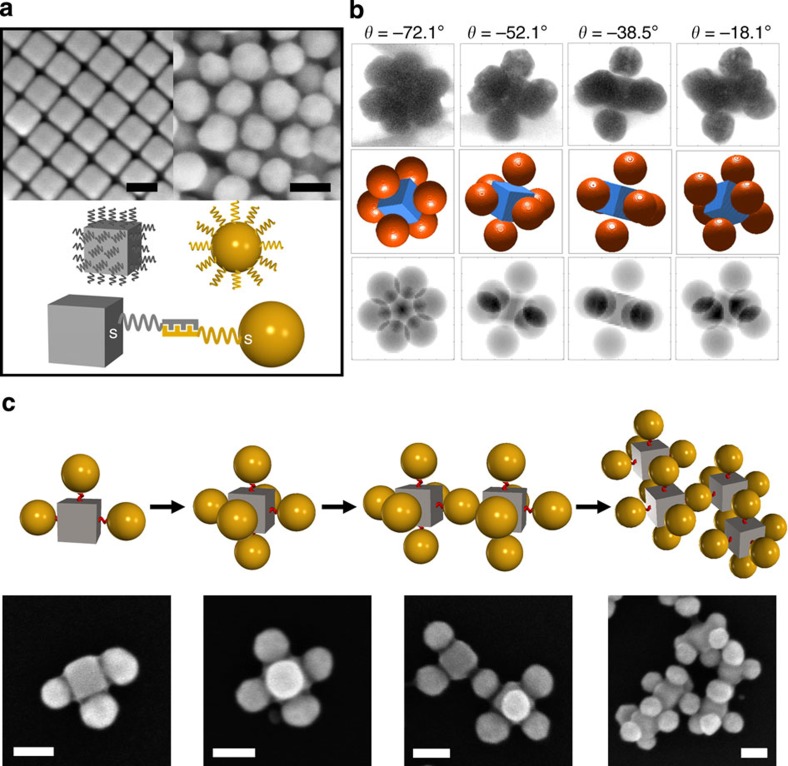
Cube-encoded assemblies of clusters with spherical nanoparticles. (**a**; Top) SEM images of (from left to right) CBs (46 nm in edge length) and SNPs (46 nm in diameter); (bottom) schematic of the DNA functionalization and assembly of CB and SNP. (**b**) Comparison of (top) TEM images with (middle) 3D reconstruction models and (bottom) the projections of the modelled structures at a few selected tilt angles to reveal the 3D structure of the assembled clusters (from left to right: –72.1°, –52.1°, –38.5° and –18.1°). The projections of the modelled clusters agree with the recorded TEM images. (**c**; Top) Schematic and (bottom) SEM images illustrate the shape-induced directional bonding of CB in a cluster-dimension-extending history (from left to right). The scale bars, 50 nm.

We first studied the binary system of spherical and polyhedral nanoparticles using gold (Au) nanosized cubes (CBs). The edge length of Au CBs and the diameter of SNPs are selected to be similar (46 nm; Fig. 2a and Supplementary Fig. 1). We coated Au CBs and SNPs with two types of complementary 18-base single-strand (DNA design denoted as *18S*) DNAs, 18A and 18A′, respectively (Fig. 2a). Each type of DNA contains a 6-base outer recognition region and a 12-base internal spacer region (see Supplementary Table 1). To study local coordination, we mixed SNPs and CBs in a number ratio of 6:1. Using transmission electron microscopy (TEM), we observed the formation of a large cluster population (∼63%) in which the central CB is surrounded by six SNPs, along with some incomplete clusters containing a smaller number of SNPs.

To reveal the 3D spatial arrangement of SNPs around the CB, we performed a TEM tomographic reconstruction. A series of TEM images from –72° to +15° (2° steps) were acquired from a typical cluster containing six of SNPs coordinated around CB. For our model-based reconstruction, we assumed that the centre particle is cubic (46 nm in edge length) and the surrounding particles are spherical (46 nm in diameter; Fig. 2b and Supplementary Note 1). The reconstruction results (Fig. 2b central panel, and the corresponding simulated projections on the bottom panel) reveal that six SNPs are positioned on the centres of the six flat facets of the CB (Supplementary Movies 1 and 2). These observations indicate that the cubic particle coordinate SNPs in accordance with a valence determined by the symmetry of CB facets. To promote an unrestricted assembly of a large-scale structure, we mixed CBs and SNPs in a number ratio of 1:1. At the initial stages of aggregation at room temperature, finite-sized clusters of various configurations were observed (two left images on Fig. 2c and Supplementary Fig. 2a). Although a broad population of CB-SNP was detected at this stage, we stress that the maximum observed coordination for CB was 6, as defined by its facet-induced valence. At the later assembly stages, the merging of clusters occurred (two right images on Fig. 2c and Supplementary Fig. 2b) and larger-scale assemblies (Supplementary Fig. 2c) were eventually formed. The geometrical constraints and local DNA-induced interactions between CBs and SNPs result in a particular arrangement of particles in the growing agglomerate (Fig. 2c and Supplementary Fig. 2). The CBs and SNPs are positioned in predominantly alternating way; however, only short-range order is realized in this arrested state because of local DNA crowding and strong interactions between complementary adhesive DNA shells.

The formation of equilibrium assemblies in DNA-guided systems requires a re-arrangement of DNA bonds formed during the initial uncontrolled aggregation. This transition can be achieved by thermal annealing, which induces partial melting (dissociation) of DNA duplexes, allowing particles to optimize their location in order to maximize the number of DNA hybridizations, that is, to satisfy an energetic criterion. For example, in the SNP/CB system, samples assembled at room temperature were subsequently annealed at a pre-melting temperature (∼39 °C) for several hours, followed by gradually cooling back to room temperature. We employed synchrotron-based small-angle X-ray scattering (SAXS) to probe *in situ* the lattice structure formation and the role of annealing temperature, *T*_a_. The peak positions and relative intensities of SAXS peaks provide insights on internal organization of assemblies in solution, while the number of peaks and their widths reflect their degree of ordering. The phase evolution from an amorphous to a crystalline state is observed as *T*_a_ approaches the melting temperature gradually (Supplementary Fig. 3). Specifically, very little change is observed until 37 °C; however, at higher temperature, the development of new higher-order peaks in the SAXS pattern, with the corresponding significant sharpening of peaks, indicates a transition to a long-range crystalline ordering.

**Figure 3:**
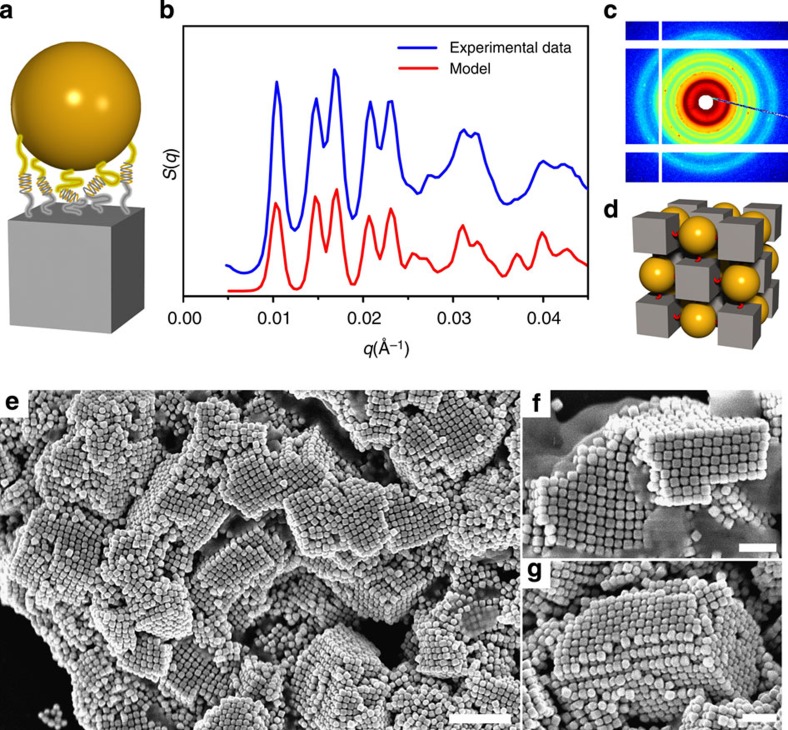
Cube–sphere nanoparticle assemblies of superlattices. (**a**) Schematic of a 46-nm SNP/46-nm CB pair linked by DNA system 18S. (**b**) SAXS data with experimental (blue) and modelled (red) structure factors, *S*(*q*), (**c**) scattering image and (**d**) the corresponding structure schematic for 46-nm SNP/46-nm CB assembly system, which crystallizes into a NaCl-type lattice with a space group symmetry of 
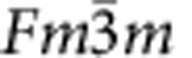
. (**e**) Low-magnification SEM image of SNP/CB-assembled crystals, where square-lattice ordering can be observed from the fragments, even though drying effects cause cracks in the crystals (scale bar, 500 nm). (**f**,**g**) High-magnification images of superlattice, demonstrating the alternate packing of SNPs and CBs in the 3D square lattice (scale bar, 200 nm).

Indeed, the annealed (at 39 °C) lattice exhibits a remarkable degree of order (Fig.3b-c) as evident from the more than 12 sharp peaks in the structure factor, *S*(*q*) (Fig.3b, blue line), for the system assembled from 18-base single-strand DNA-functionalized 46-nm CBs and 46-nm SNPs (Fig. 3a). The structure factors were obtained as *S*(*q*)*=I*_a_(*q*)/*I*_p_(*q*), where *I*_a_(*q*) and *I*_p_(*q*) are background-corrected SAXS profiles measured from the aggregate in buffer solution, and free particles in solution, respectively. The correlation length of the formed superlattice is at least 0.5 μm, estimated from the width of the first scattering peak as *ξ*=2π/Δ*q*, where Δ*q* is a resolution-corrected (Δ*q*_res_∼0.0007 Å^−1^) full-width at half maximum, and as confirmed by our detailed analysis (Supplementary Note 2). A quantitative analysis^42^ of *S*(*q*), which accounts for lattice type, particle shapes, relative particle orientations and interparticle distances (Supplementary Note 2), provides insight into the structural organization of the lattice formed by CBs and spheres.

The proposed structure, a NaCl-type lattice with space group symmetry of 
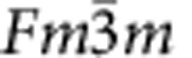
, is based on the alternating arrangement of spheres and CBs in the lattice (Fig. 3d), a symmetry imposed by the constraints of ‘*sticky*' cubic ANPs. The modelled *S*(*q*) exhibits (Fig. 3b, red line) an excellent agreement with the data (Fig. 3b, blue line), thus confirming that CBs organize SNPs into a face-centred cubic (*fcc*) lattice with a two-component basis. The position of the first *S*(*q*) peak, *q*_1_, corresponds to the {200} crystalline plane in a NaCl-lattice unit cell. There are six nearest SNPs surrounding each CB, and six nearest CB around each SNP, indicating a coordination number of 6. Such an arrangement of SNP is different from the more conventional body-centred cubic (*bcc*) lattice formed by the assembly of identical spheres with intercomplementary DNA (Supplementary Fig. 4)^3^,^4^. On the basis of the analysis (Supplementary Note 3) lattice constant, *a*, for this NaCl-type structure is 120.8 nm, which corresponds to a surface-to-surface distance between CB and SNP of *d*=14.4 nm (*d*=*a*/2–*L*_CB_/2–*D*_SNP_/2, *L*_CB_ and *D*_SNP_ are the CB edge length and the SNP diameter, respectively). Our analysis indicates that the lattice formed from CBs and SNPs exhibits not only a good translational order, but also that the CBs are oriented uniformly (Supplementary Note 2). We emphasize that the SAXS measurements are performed *in situ*, and thus represent the native, solvated configuration of the superlattice. Moreover, the SAXS data demonstrate that only a single phase is formed over the whole macroscopic dimension (∼0.2 mm) of the sample.

To further probe the superlattice structure, particularly to assess the local CB–SNP arrangements, we used direct visualization using scanning electron microscopy (SEM) after the annealed aggregates were deposited on a substrate. A low-magnification image (Fig. 3e) shows that, although drying unavoidably induces breaks in the crystallites, the long-range crystalline order is clearly evident within micron-sized domains. The domains visualized after drying in fact exhibit a specific and uniform crystal shape, cubic, the Wulff equilibrium crystal structure. High-magnification images of individual domains further demonstrate that the CBs and SNPs are arranged in an alternating manner in the 3D cubic lattice (Fig. 3f,g). One can conclude that since CBs coordinate SNPs according to the symmetry of their faces, the space group of the formed lattice is strongly correlated with the point group of CB symmetry. In particular, the CB with point-group symmetry of *O*_h_ directs the SNPs into a NaCl-type lattice with space-group symmetry of
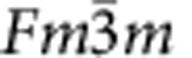
, which is a subgroup of *O*_h_.

### Effects of particle sizes and DNA shells

**Figure 4:**
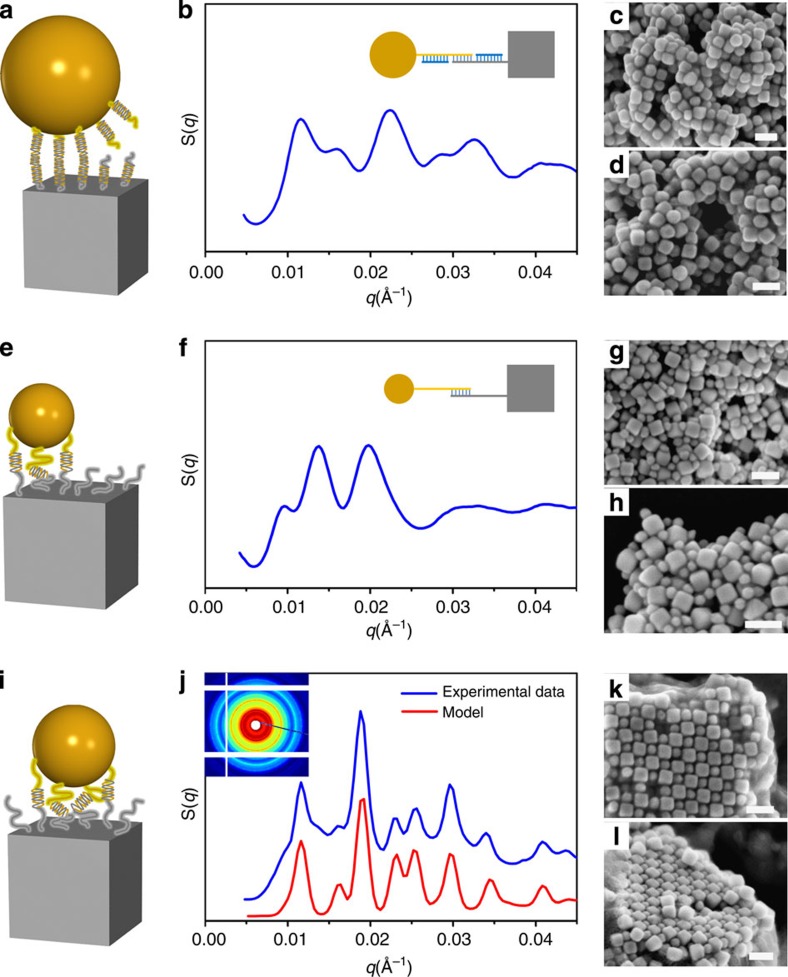
Effects of DNA rigidity and sphere size on the structure of cube-sphere binary assembly. (**a**) Schematic of a 46-nm SNP/46-nm CB pair linked by rigid DNA system 18*R*. (**b**) SAXS extracted structure factors S(*q*) and (inset) DNA-NP schematic (18*R*). (**c**,**d**) SEM image of 46-nm SNP/46-nm CB assemblies for rigid DNA system 18*R*. (**e**) Schematic of a 27-nm SNP/46-nm CB pair linked by flexible DNA system 18*S*. (**f**) SAXS data and (inset) DNA-NP schematic (18*S*). (**g**,**h**) Corresponding SEM. (**i**) Schematic of a 38-nm SNP/46-nm CB pair linked by flexible DNA system 18*S*. (**j**) SAXS data with experimental (blue) and modelled (red) structure factors, S(*q*), and (inset) scattering image, which crystallizes into a NaCl-type lattice. (**k**,**l**) Corresponding SEM images. Scale bars, 100 nm.

**Figure 5:**
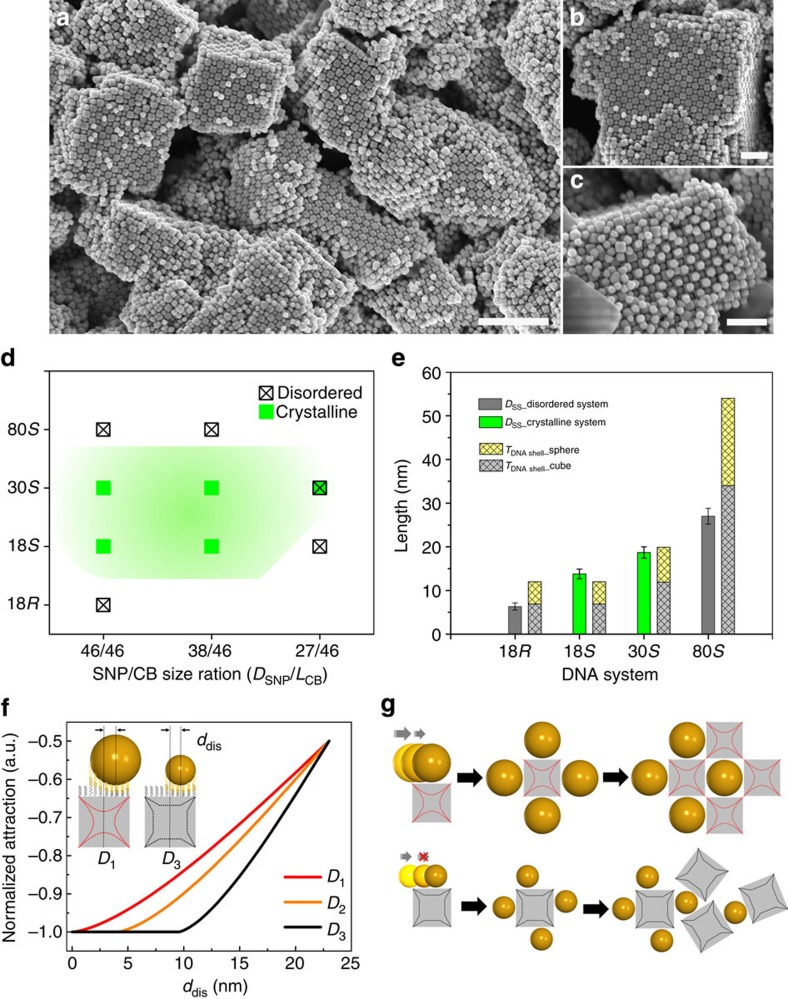
Synergetic effects of DNA shell and particle size mismatch on the structure of cube–sphere binary assembly. (**a**) Low- and (**b**,**c**) high-magnification SEM images of binary superlattices formed by 38-nm SNPs/46-nm CBs with 30*S* DNA (left scale bar, 500 nm; right scale bar, 200 nm). (**d**) State diagram for all the studied binary systems of cubes, spheres and DNA designs. Leading parameters are the DNA length and flexibility, and the SNP/CB size ratio (CB is 46 nm in edge length), as discussed in the text. (**e**) Comparison between experimental (from SAXS data) and calculated (see Supplementary Note 4) values of the nearest-neighbour interparticle surface-to-surface distances, *D*_ss_, for 46-nm SNP/46-nm CB binary assembly (error bars correspond to one s.d. for repeated experiments). The calculated values account for the sum of DNA shell thicknesses on the cube and sphere surfaces, *T*_DNA shell__cube+*T*_DNA shell__sphere. (**f**) A normalized attractive potential energy as a function of the SNP displacement, *d*_dis_, from the CB axis that crosses the centre of the square face for SNPs with diameters of 46 nm (red line *D*_1_), 38 nm (orange line *D*_2_) and 27 nm (black line *D*_3_), respectively, and schematics of SNP/CB pair for calculations (inset, left for *D*_1_ and right for *D*_3_). (**g**) Assembly of similarly sized and dissimilar CB and SNP. The mechanism for order propagation from the cluster formation of similar-sized cubes and spheres (top), and disorder (bottom) is illustrated, see the text for details.

The DNA interactions play a central role in realizing the assembly of dissimilarly shaped CBs and SNPs. This strong interaction efficiently prevents phase segregation of the two components, which would otherwise occur because of shape mismatch. We further study the effects of DNA structure and particle size regimes on the formation of ordered assemblies of CBs and SNPs. First, we alter the DNA design (*18S*), which facilitated a superlattice formation, by making it more rigid. In this new design (DNA system denoted as *18R*), the internal spacer region is an eight base-pair *duplex*. For the same 46-nm SNP/46-nm CB particle system previously described, the more rigid *18R* DNA linkages gave rise to only short-range order, even after annealing (refer to SAXS and SEM images, Fig. 4a–d). This suggests that using flexible DNA (*18S*) is essential for accommodating DNA bonds between the flat surface of CB and the curved surface of SNP. Furthermore, the polymeric nature of flexible DNA spacer strands might help masking some size disparity between CB and SNP. To examine this size-mismatch tolerance, we probed the assemblies of 46-nm CBs with two types of smaller size SNPs (while maintaining the number ratio of 1:1), 27 and 38 nm, respectively, using *18S* DNA. Size mismatches, Δ, for 27 and 38 nm SNPs, are accordingly, ∼41% and ∼17% (Δ is defined as (*L*_CB_–*D*_SNP_)/*L*_CB_). We observed the formation of only disordered morphologies for 27-nm SNPs (Fig. 4e–h), even though the same CB particles assembled with 38-nm SNPs formed a well-defined NaCl-type superlattice (Fig. 4i–l). SAXS pattern in Fig. 4j indicates about 12 sharp diffraction peaks and long-range order (correlation length ∼0.4 μm), demonstrating a good crystalline quality comparable to that of the size-matched system (46-nm SNPs/46-nm CBs, Fig. 3b). For this lattice, we obtained *a*=108.3 nm and *d*=12.2 nm; the shorter surface-to-surface distance might indicate a moderate DNA chain compression in order to compensate for the size disparity of CB and SNP. The crystalline morphology of dry 38-nm SNP/46-nm CB sample was further visualized by SEM images (Fig. 4f,g). The top layers of the assembled superlattice show a well-preserved positional and orientational order of CBs, with each relatively small spherical particle is trapped inside a square space formed by four nearest neighbour CBs. The observed tolerance of ordered lattice for particle-size mismatch was also observed in systems with longer DNA strand and flexible spacer (Fig. 5a–c). DNA system 30S consists of complementary 30-base DNAs, with each containing an 8-base outer recognition region and a 22-base internal spacer region. As shown in Figure 5a, a large-scale organization with long-range order was observed for the 38-nm SNP/46-nm CB assemblies linked by 30S DNA. Single-domain grains with up to micron-scale sizes have a cubic shape and relatively flat facets (Fig. 5b). CBs (46 nm) exhibit a remarkable orientational correlation and even enable isolating 38-nm SNPs on the top of layers (Fig. 5c).

We further investigated how the DNA structure and SNP size affect the shape-directed CB coordination of spheres, which is signified by the structure of 3D assembly. In Fig. 5d, we summarize the phase diagram with leading parameter being the DNA design and the SNP/CB size ratio for all the studied binary systems with 46-nm CB size. Several important conclusions can be drawn about the assembly of the presented heterogeneously shaped nanoparticles: (i) DNA structure and SNP size control assembly morphology, which varies from disordered aggregates to ordered lattices; (ii) flexible DNA motifs are essential for achieving crystalline organization (Supplementary Fig. 5), whereas the size match between dissimilar nano-objects is favourable but not strictly requisite; (iii) flexible DNA bridges are beneficial to the crystallization of size-mismatch SNP/CB assemblies (Figs 4i–l and 5a–c and Supplementary Fig. 6); (iv) DNA with excessively long flexible motifs results in short-range ordering even for size-matched particles (80S in Supplementary Fig. 5). The DNA shells comprising long and flexible chains mask the geometry; thus, the necessary plasticity of the interparticle connections can, if overemphasized, destroy the directional interactions provided by CBs, resulting in disordered assemblies. Furthermore, we observed that asymmetric shells, wherein the CBs and spheres were functionalized with DNA of different lengths while preserving total linker length within the crystalline zone (Fig. 5d), can induce yet more complex behaviour because of interplay of directional binding and polymeric effects (Supplementary Fig. 7).

The curved surfaces of SNPs restrict the number of DNA hybridizations that can occur without chain deformation, in comparison with interactions between flat surfaces. Indeed, DNA chains bridging the flat and curved surfaces can only maximize the number of bridges if some chains are stretched or compressed. For the size-matched NP assemblies (46-nm SNPs/46-nm CBs), the degree of DNA chain deformation, which depends on DNA structure (from 18*R* to 18*S*, 30*S*, 80*S*), can be correlated with the state behaviour (Fig. 5d). In Fig. 5e, we compared the surface-to-surface distances, *D*_ss_, in binary CB/SNP systems. We estimated *D*_ss_ as the sum of DNA shell thicknesses on two types of surfaces, *T*_DNA shell__cube+*T*_DNA shell__sphere (Supplementary Note 4). We found that the observed *D*_ss_ for the crystalline systems (18*S* and 30*S*) agrees well with our models, whereas the disordered systems (18*R* and 80*S*) exhibit larger deviations. This suggests that DNA linkers that are too rigid or too long experience severe deviations from their equilibrium conformations, with a correspondingly greater energy penalty. Suitable DNA designs can properly balance the compression and stretching of DNA polymer chains confined between the differently curved surfaces of CB and SNP; thereby enabling the formation of well-defined crystalline nanoparticle assemblies.

The observed influence of the size mismatch (between SNP and CB linked by 18*S* DNA) on lattice order can be understood by considering the details of interactions between the CB and SNP surfaces. The binding of SNPs in the middle of a CB's facet is an important factor for the order development, since it allows ‘fixing' the subsequent attachments in a symmetric manner. SNP positioning in the middle of a CB face is favourable when Δ is small. Indeed, the adhesive energy between SNP and CB is proportional to the number of DNA chains hybridized between them, which can be approximated as the projected area of the SNP on the square face of the CB^43^. If the SNP diameter is smaller than the CB's edge length, then the SNP placement becomes ill-defined. In this case, the adhesive energy (approximately proportional to the number of DNA bridges) is equivalently maximized over a broad range of SNP positions on the CB face. In contrast, if the SNP and CB are comparable in size, the placement in the middle is uniquely favourable because of maximized adhesion (free energy minimized). This difference between a large free energy plateau and a well-localized minimum is essential for defining the particle position on the CB face. We calculated the attraction energy as function of the SNP displacement, *d*_dis_, from the CB central axis crossing the CB's face centre (Fig. 5f and Supplementary Note 5). Decreasing the SNP's diameter results in an increase in the attractive potential plateau. The attraction potential curves between SNP and CB are schematically visualized by overlapping with CB shapes, as shown by the in-square red and black lines in Fig. 5f,g.

Figure 5g illustrates the influence of this effect on lattice order. For matching sizes of SNP and CB, the SNPs are constrained into a very narrow equilibrium position on the CB's face, which promotes the alignment of CBs and SNPs over long ranges. In contrast, spread in the position of the small SNP on the face results in a random attachment of subsequent CBs and, consequently, in lattice disorder. However, as discussed above, a larger degree of mismatch can be compensated by longer chains of single-stranded DNAs (that is, DNA system 30*S*).

### Assembly of octahedron and sphere nanoparticles

**Figure 6:**
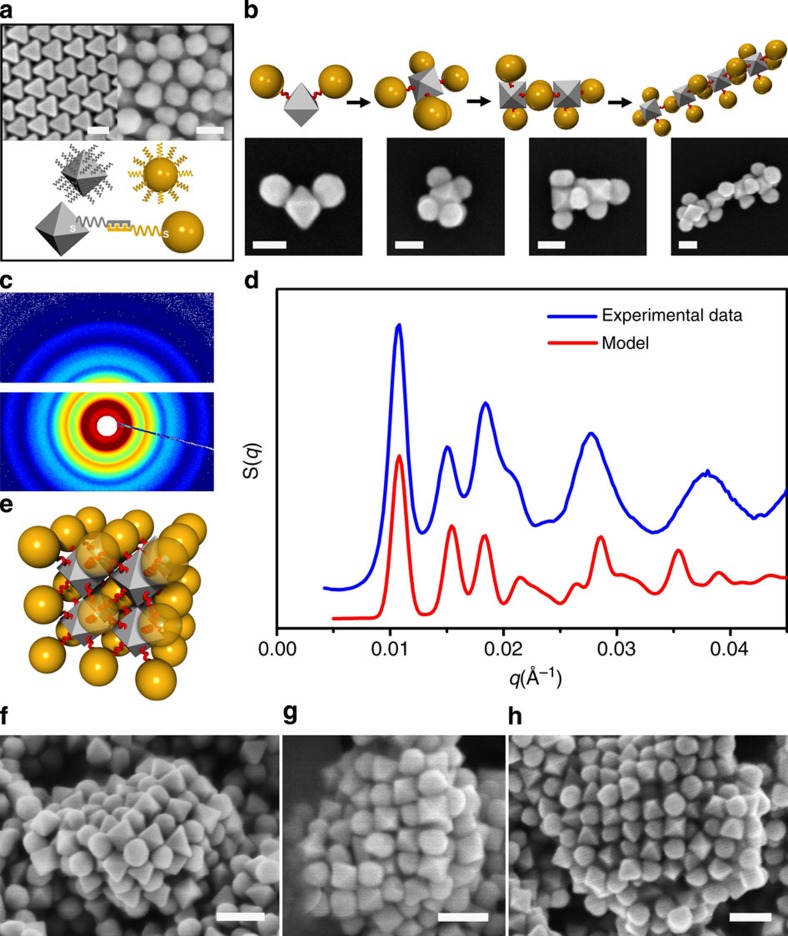
Octahedron-sphere nanoparticle assemblies. (**a**; Top) SEM images of (from left to right) OCs (46 nm in edge length) and SNPs (46 nm in diameter); (bottom) schematic of DNA functionalization and assembly of OC and SNP. (**b**; Top) Schematic and (bottom) SEM images illustrating the directing role of octahedral blocks in the formation of clusters, and their merging into larger-scale structures (from left to right). Scale bars, 50 nm. (**c**) SAXS image and (**d**) data with experimental (blue) and modelled (red) structure factors, *S*(*q*). (**e**) The corresponding structure of formed CsCl-type superlattice with space group 
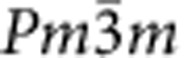
 symmetry (left bottom) for 46-nm SNP/46-nm OC assemblies. (**f**–**h**) SEM images of SNP/OC-assembled superlattice fragments (scale bar, 100 nm).

We further studied the assembly of heterogeneously shaped particles by choosing octahedron (OC) as anisotropic particle to assemble with spheres (Fig. 6a). The OC is a dual pair of the CB, and belongs to the same symmetry group, but exposes eight triangular faces. We selected Au OCs and SNPs of ∼46 nm in the edge length and diameter, respectively. OCs and SNPs were functionalized with two types of complementary 50-base single-strand DNAs, 50A and 50A′, respectively (Fig. 6a and see Supplementary Fig. 1 and Supplementary Table 1), with each type of DNA containing an eight-base outer recognition region. The OC via its triangular facets provides eightfold coordination for SNPs. The SEM imaging indeed shows that OCs effectively coordinate spheres initially to some, and then to all, of the eight faces (Fig. 6b). Analogous to the CB's case, clusters formed by coordinating spheres around OCs merge into larger aggregates, in which OCs and SNPs alternate (Fig. 6b and Supplementary Fig. 8).

The *in situ* SAXS measurements (Fig. 6c) demonstrate that the OCs and SNPs form a well-defined CsCl-type lattice, in which the OCs retain their orientational order, as evident from the experimental and modelled structure factors (blue and red curves, respectively, in Fig. 6d), which are in a good agreement (Supplementary Note 2). This *bcc* type phase (*a* of ∼82 nm and domain size of ∼0.4 μm) with a two-component basis can also be viewed as two interpenetrating simple cubic (*sc*) lattices, wherein OCs and SNPs define the lattice sites of each sublattice (Fig. 6e). The *q*_1_ position in the SAXS data corresponds to the {110} crystalline planes in a CsCl-type lattice (Fig. 6e and Supplementary Note 3). In this lattice, eight SNPs surround each OC, and eight OCs surround each SNP, that is, the coordination number is 8, which is reminiscent of perovskite crystals. SEM provides additional information about the arrangement of OCs and SNPs (Fig. 6f–h and Supplementary Fig. 9). Square domains and alternating shapes can be distinguished in the low-magnification large-area image (Supplementary Fig. 9a). High-magnification images confirm that the SNPs are attached to the triangular facets of OC; the alternating arrangement of OCs and SNPs in a CsCl-type lattice is clearly observed (Fig. 6f–h and Supplementary Fig. 9b–e).

## Discussion

Notably, these results demonstrate that, despite having the same *O*_h_ point-group symmetry as CBs, the OCs, due to their facet arrangement, direct SNPs into a different lattice. The OCs yield CsCl-like lattices (*bcc*), whereas the CBs produce NaCl-like (*fcc*) crystals. Both 
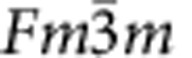
 (NaCl) and 
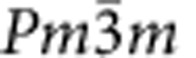
 (CsCl) are subgroups of the *O*_h_ point group, to which our polyhedral ANPs (CB and OC) belong, and in which they define a dual pair. However, as we observed, the formation of the specific lattice type from the group requires a particular symmetry of coordination of SNPs, and that is provided by the shape of the ANPs via its adhesive facets. To satisfy this seemingly simple rule, as we show, is not trivial, because of the effects of different local curvatures and interactions of dissimilarly shaped nano-objects. These effects play a crucial role in the formation of ordered assemblies. Hence, for optimized interaction in these lattices, the highest-order symmetry object, the sphere, is fully constrained by the low-order symmetry object, the anisotropic polyhedron.

In summary, we demonstrate that directional bonds provided by polyhedral nanoparticles can be used effectively for arranging isotropically interacting spherical nanoparticles into 3D superlattices. We uncover the primary requirements for the geometrical features of polyhedral blocks to be robustly translated into 3D crystalline order; namely, appropriate tuning of DNA interactions and particle size regimes. Moreover, the origin of global lattice symmetry can be traced back to the geometry of the anisotropic blocks. The softness of particle coronas is crucial for accommodating mismatched shapes in the lattice, however, excessive flexibility results in disordered states. Furthermore, the relative sizes of dissimilar particles are imperative; long-range-ordered lattices only form when particles assemble so as to locally constrain positional order. The presented work should permit more broadly for the realization of designed hetero-assemblies, using directional interactions of anisotropic nanoblock and DNA programmability, and it will stimulate fundamental studies on the relationship between building block geometry and global crystallographic symmetry. The role of the particle's soft shell cannot be overstated. Corona softness is crucial to the formation of an accommodating interface between the differently shaped particles, thereby promoting ordered phases despite conflicting directional interactions. An extensive variety of nanoscale polyhedral objects have become available, such as tetrahedron, decahedron and icosahedron^27^,^44^,^45^,^46^; simultaneously, DNA-coating approaches have been extended to many different nanomaterial types^3^,^47^,^48^. The demonstrated approach will thus open novel opportunities for the rational design of 3D nanostructured materials from a broad variety of functional nanocomponents.

## Methods

### Synthesis of polyhedral gold nanoparticles

*Reagents:* Gold (III) chloride trihydrate (HAuCl_4_·3H_2_O, 99.9+%), sodium borohydrate (NaBH_4_, 99.99%), L-ascorbic acid (AA, 99+%), cetyltrimethylammonium bromide (CTAB, 99.9%) and cetylpyridinium chloride (CPC, 99%) were purchased from Sigma-Adrich and used without further purification. Milli-Q water with a resistivity greater than 18.0 MΩ cm was used in the preparation of aqueous solutions. *Synthesis of Au nano-octahedral seeds:* The Au nano-octahedral seeds were prepared using a modified two-step procedure^49^. First, 3-nm Au seeds were prepared by quickly injecting 0.60 ml of ice-cold, freshly prepared NaBH_4_ (10 mM) into a rapidly stirred mixture of HAuCl_4_ (10 mM, 0.25 ml) and CTAB (0.1 M, 9.75 ml). The seed solution was stirred for 2 min and then left undisturbed at 25 °C for 3 h to ensure complete decomposition of NaBH_4_ remaining in the solution. The seed solution was diluted 100 times with 0.1 M CTAB. For the synthesis of octahedral Au seeds, 4 ml of aqueous HAuCl_4_ solution (0.3 mM), 24 ml of aqueous CTAB solution (20 mM) and 1.8 ml of aqueous AA solution (100 mM) were mixed, followed by adding 360 μl of the diluted seed solution under stirring. The mixture solution was then left to age at 25 °C for 12 h. *Seed-mediated growth of polyhedral Au nanoparticles:* In a typical synthesis of the Au nanocubes, 5 ml of the above 12-h-aged seeds was first washed three times with 0.1 M CTAB solution using centrifugation (10,000 r.p.m., 10 min) and finally condensed in 500 μl of 16 mM CTAB solution; 100 μl of 10 mM HAuCl_4_ solution, 400 μl of freshly prepared 100 mM AA and 500 μl of the condensed seed solution were added to 5 ml of 16 mM CTAB solution at 32 °C consecutively and thoroughly mixed after each addition. The reaction was then left undisturbed for 6 h and stopped by centrifugation (10,000 r.p.m., 10 min). The obtained nanoparticle solution was washed twice with Milli-Q water and concentrated for further usages. In a typical synthesis of the Au OCs, 5 ml of the above 12-h-aged seeds was first washed three times with 0.1 M CPC solution by centrifugation (10,000 r.p.m., 10 min) and finally condensed in 500 μl of 0.1 M CPC solution; 100 μl of 10 mM HAuCl_4_ solution, 13 μl of freshly prepared 100 mM AA and 500 μl of the condensed seed solution were added to 5 ml of 0.1 M CPC solution at 25 °C consecutively and thoroughly mixed after each addition. The reaction was then left undisturbed for 6 h and stopped by centrifugation (10,000 r.p.m., 10 min). The resultant nanoparticle solution was washed twice with Milli-Q water and concentrated for further usage. Average edge lengths of the synthesized Au nanocubes and nano-OCs are similar, being of ∼46 nm. Au nanocubes have a size distribution s.d. of ∼2% and a yield of ∼96%; Au nano-OCs have a size distribution s.d. of ∼3% and a yield of ∼95%. The results were obtained from counting at least 200 nanoparticles for each sample.

### Nanoparticle functionalization with DNA

Thiol-modified single-strand oligonucletides (see Supplementary Table 1 for sequences) were purchased from Integrated DNA Technologies Inc. with disulfide modification. Before nanoparticle DNA functionalization, the disulfide oligonucleotides were first reduced by dissolving the lyophilized samples (100∼300 nmoles) in 0.3 ml of a 100-mM dithiothreitol (DTT) solution in purified water or buffer. The reduced DNA was loaded on a freshly purified sephadex column (G-25, Amersham Bioscience) and eluted with 2.5 ml of 10 mM phosphate buffer (pH=7.4). The DNA was quantified using UV–vis analysis using the known extinction coefficient. Au nanoparticles were functionalized with ssDNA following a previously demonstrated method to achieve high DNA coverage^32^. Briefly, an aliquot of purified DNA solution was added to 1 ml aliquot of Au nanoparticles (∼3 OD_260_ of DNA for per ml of nanoparticle colloid). After allowing 1–3 h for thiolated DNAs to react with the gold surface, particle suspensions were brought to 0.01% sodium dodecyl sulfate (SDS) and 10 mM sodium phosphate and allowed to sit for 1 h. Following literature procedures, the colloidal nanoparticle solutions were then slowly treated with NaCl to allow for electrostatic screening between neighbouring DNA strands and denser surface coverage of oligonucleotides. Specifically, NaCl concentration of the solution was brought to 0.5 M slowly by adding aliquots of 3 M NaCl eight times with ∼30-min interval for incubation. After reaching the final NaCl concentration, particles were allowed to sit overnight to achieve maximum DNA loading. To remove the excess, unbound DNA from the solution, the mixture was centrifuged, the supernatant was removed and the pellet was resuspended in washing buffer (0.01% SDS+10 mM phosphate buffer, pH=7.4). This process was repeated three times. The final resuspension typically occurred in 50–100 μl to allow for a concentrated solution of particles in 0.2 M PBS buffer (0.2 M NaCl+10 mM phosphate buffer, pH=7.4) for further assemblies. Concentration of nanoparticles was quantified using the absorbance value at the surface plasmon resonance (SPR) maximum in UV–vis absorption spectra. A molar extinction coefficient of 3.5 × 10^10^ M^−1^ cm^−1^ at 546 nm SPR peak is used for the nanocubes with ∼46 nm edge. A molar extinction coefficient of 2.5 × 10^10^ M^−1^ cm^−1^ at the 564 nm SPR peak is used for the nano-OCs with ∼46 nm edge. For the spherical Au nanoparticles purchased from Nanopartz Inc., molar extinction coefficients of 10.5 × 10^9^ M^−1^ cm^−1^ at the 532 nm peak, 5.93 × 10^9^ M^−1^ cm^−1^ at 529 nm peak and 3.6 × 10^9^ M^−1^ cm^−1^ at 527 nm are used for the nanospheres with the diameters of 46, 38 and 27 nm, respectively.

### Assembly and crystallization of nanoparticle superlattices

The assembly was obtained by combining equal molar amounts of type-A and type-A′ DNA-functionalized Au nanoparticles, and the particles were allowed to aggregate at room temperature. The samples were then annealed at a temperature ∼1–2 °C below the melting temperature of the assembled particles for a period of several hours, depending on the sample. The annealed samples were transferred with buffer to a quartz capillary (1.0 mm diameter), and sealed with wax for SAXS measurements.

### Characterization of nanoparticles and assemblies

The morphology of nanoparticles and the *ex situ* structure of assemblies were characterized using electron microscopy. Synchrotron-based SAXS (NSLS X9) was used to probe the *in situ* structure of particle assemblies. *UV-Visible Spectrophotometry (UV–vis):* UV–vis spectra were recorded on a Perkin-Elmer Lambda 35 spectrometer (200–700 nm). Melting analysis was performed in conjunction with a Perkin-Elmer PTP-1 Peltier Temperature Programmer between 20 and 75 °C with a temperature ramp of 1 °C min^−1^ while stirring, in a 10 mM phosphate buffer, 0.2 M NaCl, pH=7.4, buffer solution. *SEM:* SEM experiments were carried out on Hitachi S-4800 Scanning Electron Microscopy with typical 1 kV voltage and 10 μA emission current. The sample was prepared by drop-casting an aqueous nanoparticle solution on a cleaned silicon substrate. *TE):* TEM images were collected on a JEOL-1400 microscope operated at 120 kV. The samples were prepared by drop-casting an aqueous nanoparticle solution on a carbon-coated copper grid. *Small-angle X-ray scattering (SAXS):* SAXS experiments were performed *in situ* at the X9 beamline at the National Synchrotron Light Source. The scattering data were collected with a Dectris Pilatus 1 M pixel-array detector and converted to 1D scattering intensity versus wave vector transfer, *q*=(4*π*/*λ*) sin (*θ*/2), where *λ*=0.9184 Å, and *θ*, are the wavelength of incident X-ray beam and the full scattering angle, respectively. The data are presented as the structure factor *S*(*q*), which was calculated as *I*_a_ (*q*)/*I*_p_(*q*), where *I*_a_ (*q*) and *I*_p_(*q*) are background-corrected 1D scattering intensities extracted by angular averaging of detector images for a system under consideration, and the corresponding unaggregated gold particles, respectively. The peak positions in *S*(*q*) are determined by fitting to a Lorentzian function.

### Modelling of SAXS profiles and DNA structure

To simulate powder SAXS profiles, we used our recently published scattering formalism, which simulates powder SAXS profiles for lattices of particles with arbitrary shape^42^. This formalism accounts for particle size, particle shape and particle orientation within the unit cell. We also explicitly included disorder: particle size polydispersity, lattice disorder (Debye–Waller factor) and average grain size. We used a polyelectrolyte-blob model and a Daoud-Cotton (DC) blob model to calculate the tethered DNA thickness on a flat surface (for cube) and a curved surface (for sphere), respectively. All SAXS and DNA modelling details are provided in the Supplementary Note 2.

### SEM and layer-by-layer sample preparation

The sample deposited on a cleaned silicon substrate was measured using a Hitachi S-4800 Scanning Electron Microscope with typical 1-kV voltage and 10-μA emission current. A standard polyelectrolyte-assisted layer-by-layer (LBL) method was applied to prepare the diluted nanoparticle-assembled clusters for SEM characterization^50^. Silicon wafers were used as substrates for SEM characterization. The substrates were sonicated for 10 min in water and then in ethanol, subsequently thoroughly cleaned using piranha solution (H_2_SO_4_:H_2_O_2_=7:3), rinsed with deionized water and dried under an air stream. The wafers were stored in water before being use. The wafers were first immersed in an aqueous solution of positively charged poly (diallyldimethylammonium chloride) PDDA (Mw=200,000, 1 mg ml^−1^ in 0.5 M NaCl aqueous solution) for 20 min, and then in an aqueous solution of the polyanion poly(acrylic acid, sodium salt) PAA (Mw=15,000, 1 mg ml^−1^ in 0.5 M NaCl aqueous solution) for 10 min and finally in PDDA solution for 10 min. At this stage, the wafers are positively charged, favouring the electrostatic interaction with negatively charged DNA in the assembled aggregates. To obtain a monolayer of nanoparticle-assembled clusters, the pretreated wafers were immersed into the corresponding solution with diluted aggregates and kept for a suitable period time. After enough absorption, the substrates were rinsed with deionized water and dried under an air stream for further SEM characterization.

### Quantification of DNA grafting

A fluorescence-based method was used to determine the number of DNA loaded on particle surfaces^51^. First, the DNA was chemically displaced from the nanoparticle surface using DTT. The displacement was initiated by adding equal volumes of DNA-functionalized nanoparticles and 1.0 M DTT in 0.1 M PBS, pH=7.4. The oligonucleotides were released into solution during overnight incubation, and the particle precipitate was removed by centrifugation. The concentrations of oligonucleotide in solution were determined using fluorescence spectroscopy. During the fluorescence measurement, the fluorophore was excited at 450 nm and the emission was collected from 520 to 640 nm.

## Author contributions

F.L. and O.G. conceived the concept and designed the experiments. F.L. performed the experiments. F.L. and O.G. analysed the data. K.G.Y. performed SAXS modelling and analysis. Y.G.Z. contributed to the DNA modelling and SAXS analysis. H.L.X. contributed to TEM characterization and tomographic reconstructions. F.L. and O.G. wrote the paper. All authors discussed the results and commented on the manuscript.

## Additional information

**How to cite this article**: Lu, F. *et al*. Superlattices assembled through shape-induced directional binding. *Nat. Commun*. 6:6912 doi: 10.1038/ncomms7912 (2015).

## Supplementary Material

Supplementary Information Supplementary Figures 1-9, Supplementary Table 1, Supplementary Notes 1-5 and Supplementary References

Supplementary Movie 1 TEM tilt series. The movie shows the TEM tilt series of the cube-directed nano-cluster.

Supplementary Movie 2 3D visualization. The movie shows the reconstructed nano-cluster viewed from different angles.
