# Supplementary material for: Superlattices assembled through shape-induced directional binding
Source: Nat Commun. 2015 Apr 23;6:6912. doi: 10.1038/ncomms7912 (PMC4423233; doi:10.1038/ncomms7912)
Supplement: Supplementary Information — Supplementary Figures 1-9, Supplementary Table 1, Supplementary Notes 1-5 and Supplementary References [file ncomms7912-s1.pdf]

Supplementary Figure 1

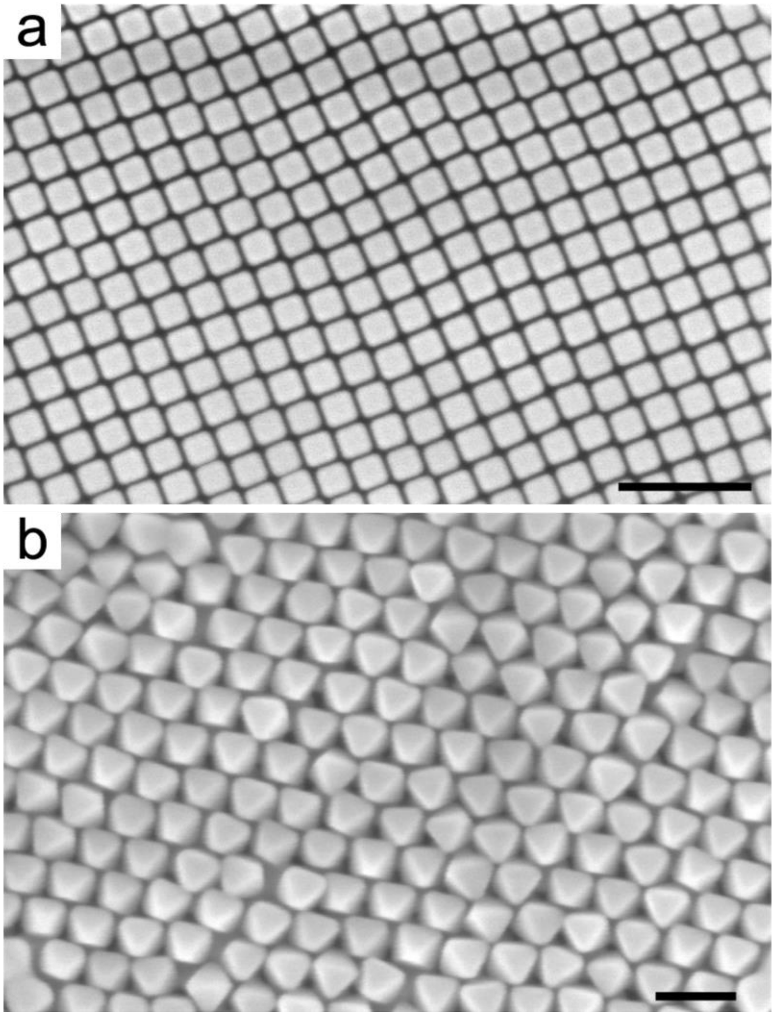

Supplementary Figure 1. SEM images of Au nano-polyhedrons. (**a**) Cubes (CBs, scale bar: 200 nm) and (**b**) Octahedrons (OCs, scale bar: 100 nm).

Supplementary Figure 2

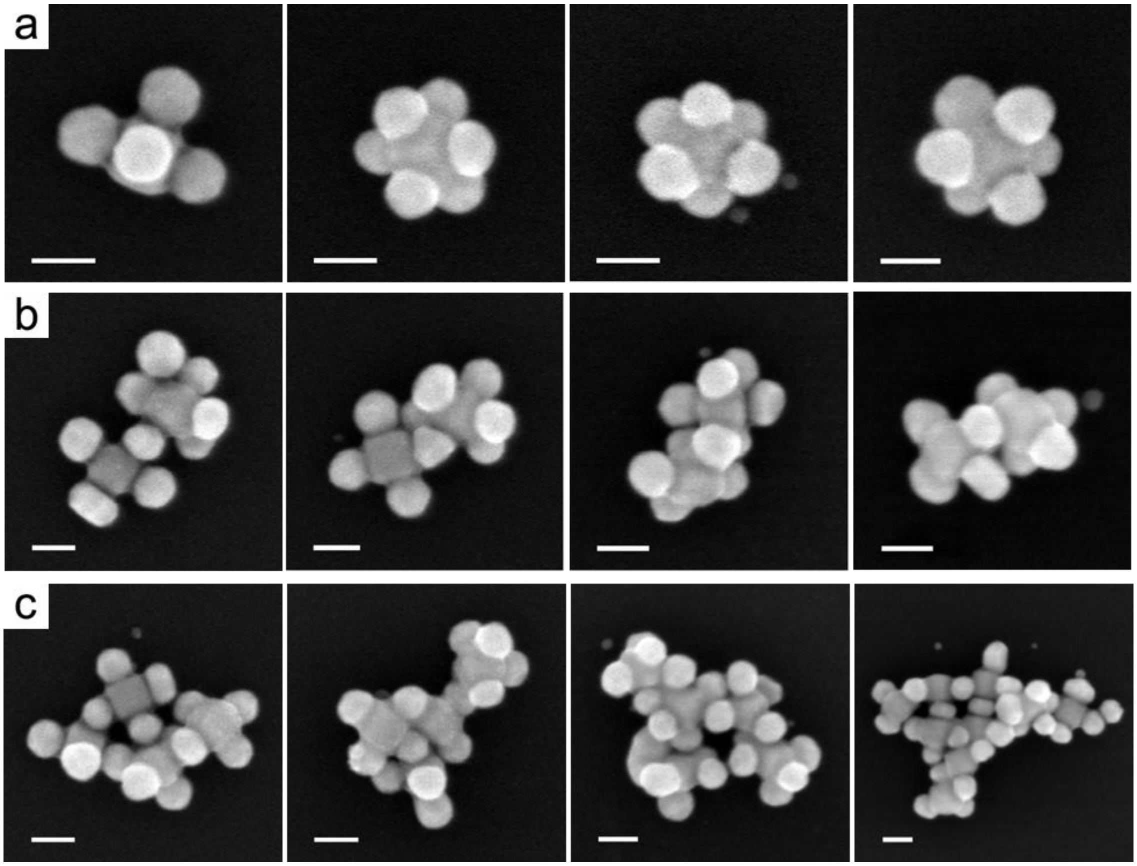

Supplementary Figure 2. Cube-encoded assemblies of clusters. Representative SEM images illustrating evolution stages (from **row a, b,** to **c**) of the clusters assembled from the 46 nm SNPs/46 nm CBs binary-shape systems. Scale bars: 50 nm.

Supplementary Figure 3

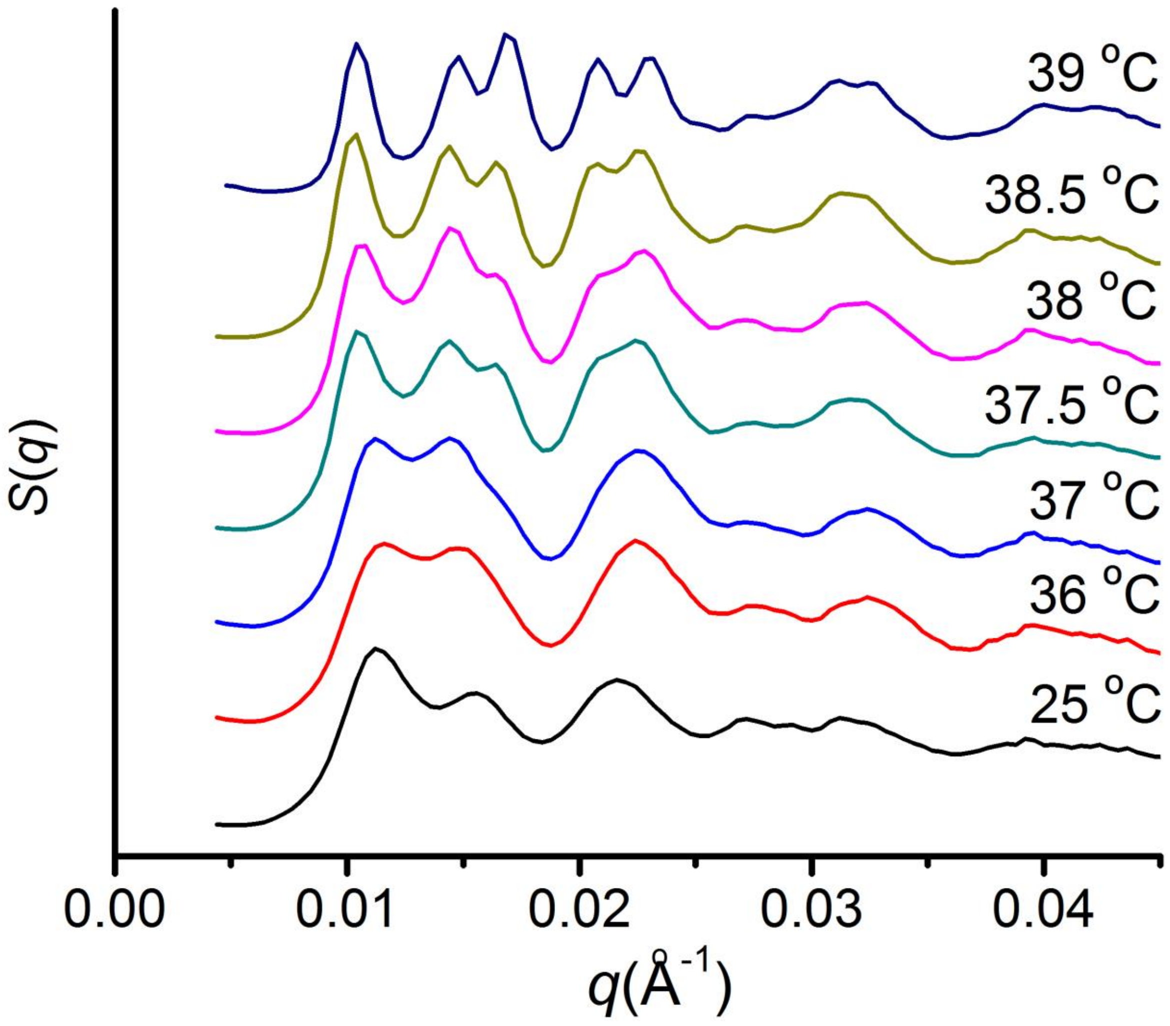

Supplementary Figure 3. Effect of annealing temperature on crystallization. Crystallization pathway for the 46 nm SNPs/46 nm CBs assemblies linked by DNA system 18*S*. SAXS extracted structure factors $S(q)$ as a function of the annealing temperatures (T_a) shown.

Supplementary Figure 4

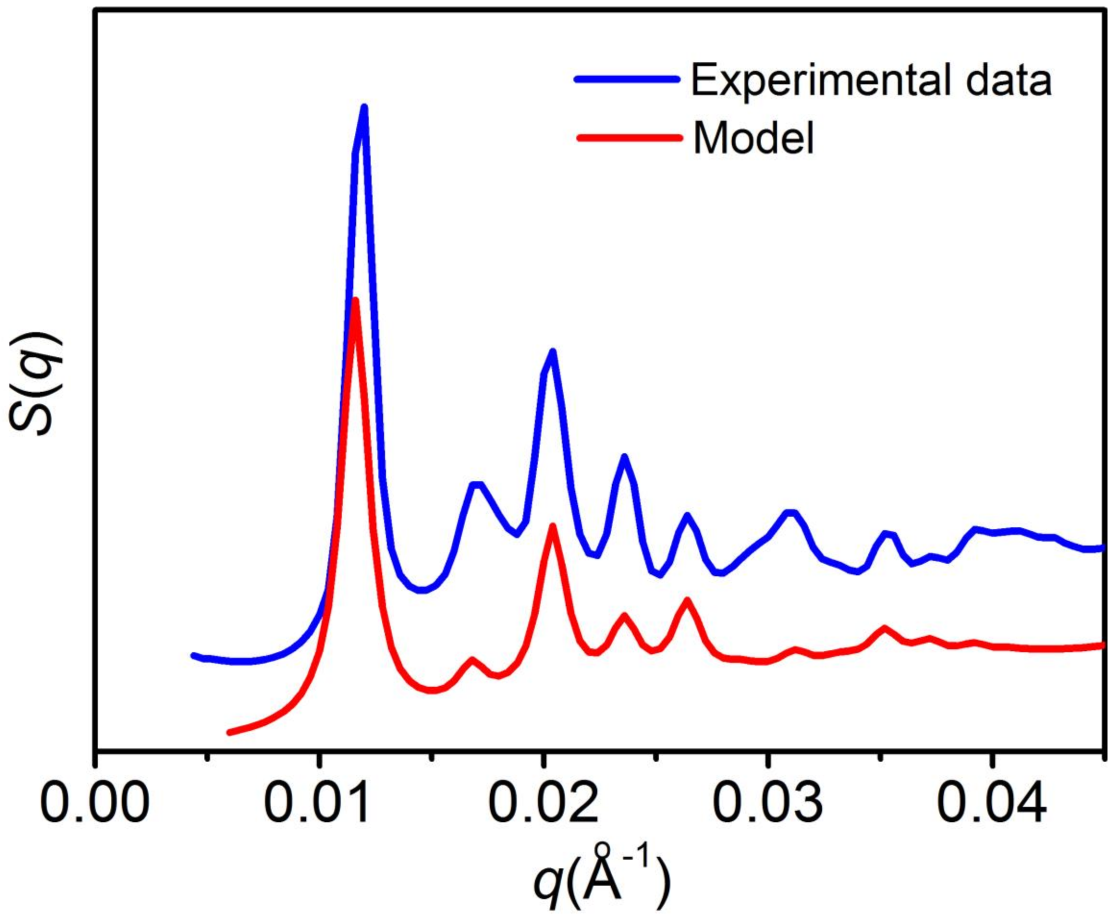

Supplementary Figure 4. DNA-mediated assembly of 46 nm spherical nanoparticles. Experimental (blue) and modeled (red) SAXS patterns for the mono-component assemblies of spherical particles: 46 nm SNPs crystallize into a *bcc* lattice (DNA_A=5'-HS-C_6H_{12}-$(T)_{15}$-TAACCTAACCTTCAT-3'; DNA_A'=5'-HS-C_6H_{12}-$(T)_{15}$- TAACCTAATGAAGGT-3').

Supplementary Figure 5

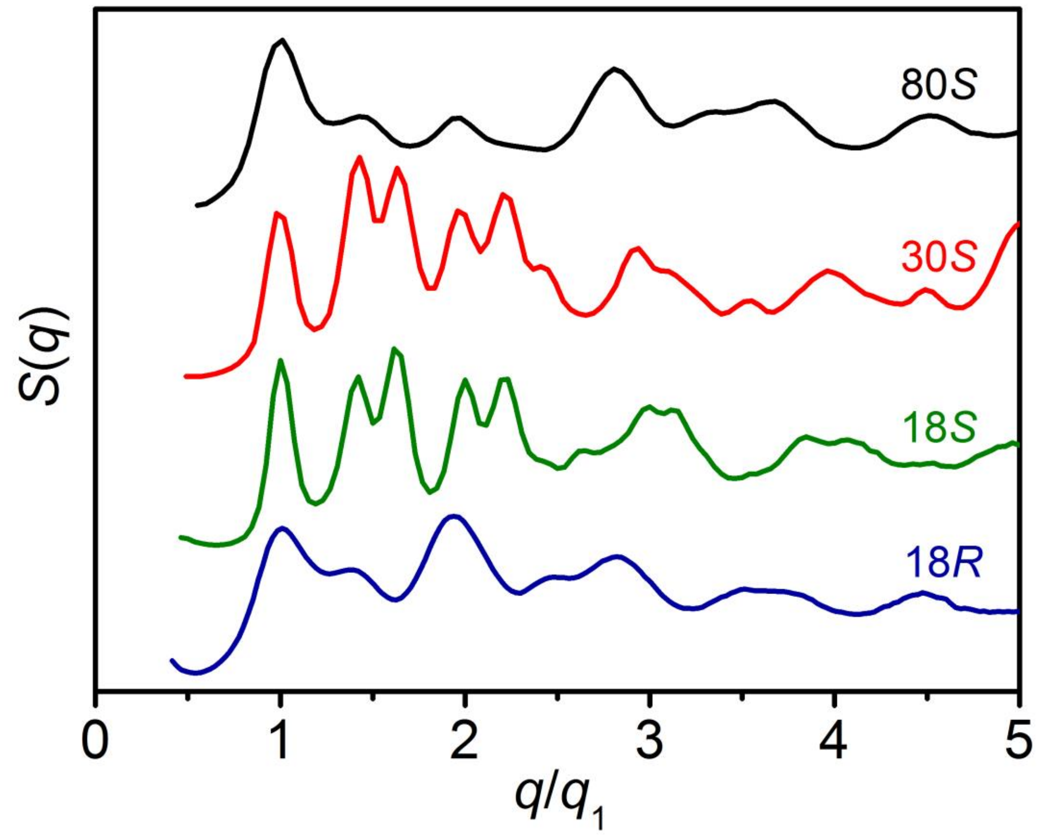

Supplementary Figure 5. Effect of DNA shells on crystallization. Comparison of SAXS patterns for the 46 nm SNPs/46 nm CBs binary assemblies linked by different DNA systems. The 18*R* and 50*S* samples formed disordered structures while the 18*S* and 30*S* samples crystallized into NaCl-type lattices.

Supplementary Figure 6

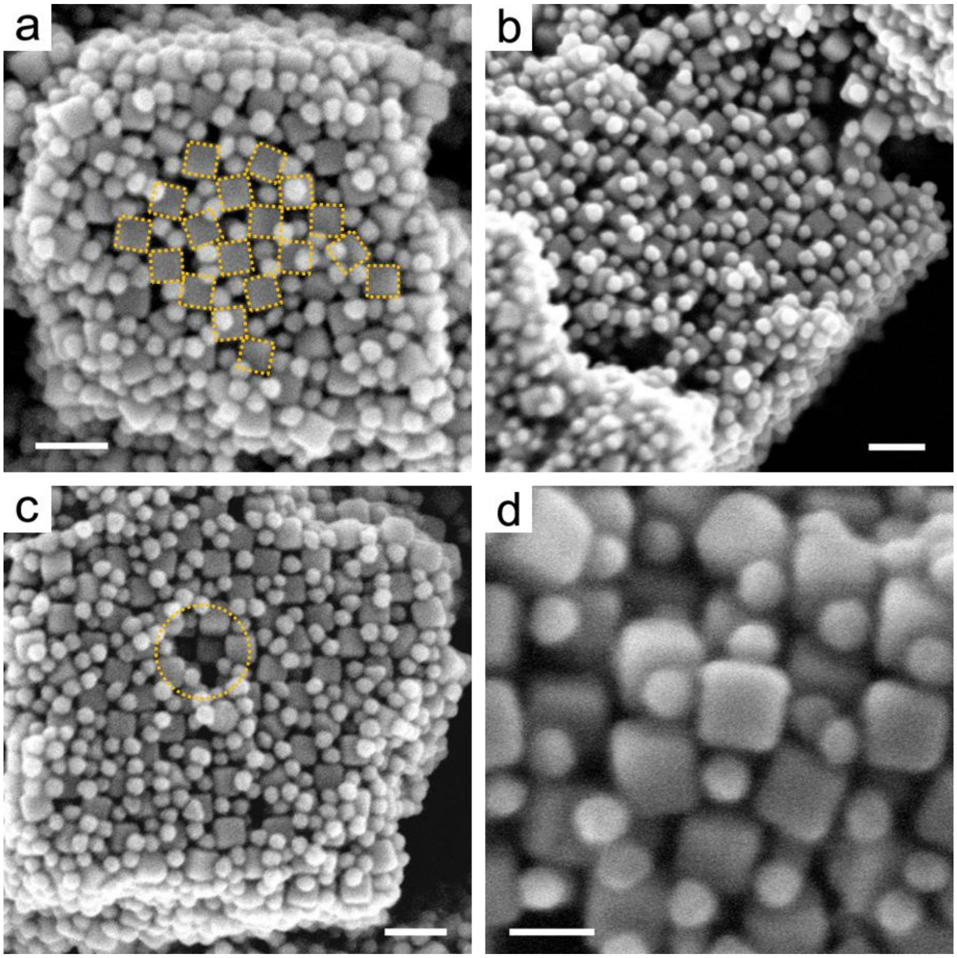

Supplementary Figure 6. Morphology of the 27 nm SNPs/46 nm CBs assemblies. Representative SEM images of the 27 nm SNPs/46 nm CBs binary assemblies linked by DNA system 30*S*. DNA system with longer spacers can compromise a larger size mismatch between SNP and CB. Compared to the fully disordered assemblies (DNA 18*S*_27 nm SNPs/46 nm CBs, Fig. 4g-h), the orientation order of CBs here was preserved to some extent after dry-induced collapse. Scale bars represent 100 nm in **a-c** and 50 nm in **d**, respectively.

Supplementary Figure 7

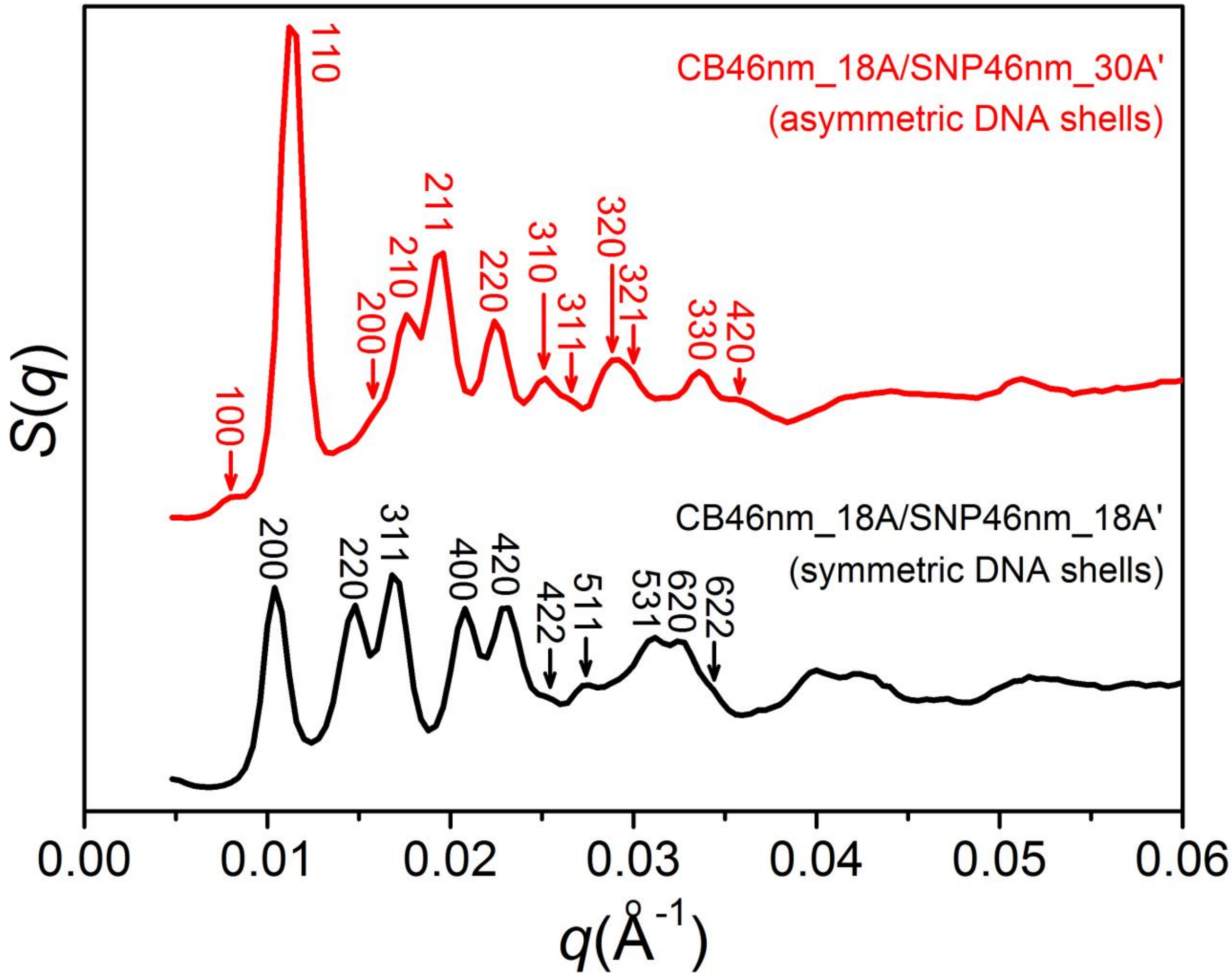

Supplementary Figure 7. Effect of DNA shells symmetry on particles assembly. The effect of DNA shells symmetry on the final crystalline structures of 46 nm CBs/46 nm SNPs binary assemblies. (**Top**) red line: SAXS pattern for the assembly with an asymmetric design of DNA shells (the base numbers of the DNA grafted on CB and SNP are 18 and 30, respectively, with a length difference of ~2 nm), which is indexed as a CsCl-type structure. (**Bottom**) black line: SAXS pattern for the assembly with a symmetric design of DNA shells (the base numbers of the DNA grafted on CB and SNP are both 18), which is indexed as a NaCl-type structure.

Supplementary Figure 8

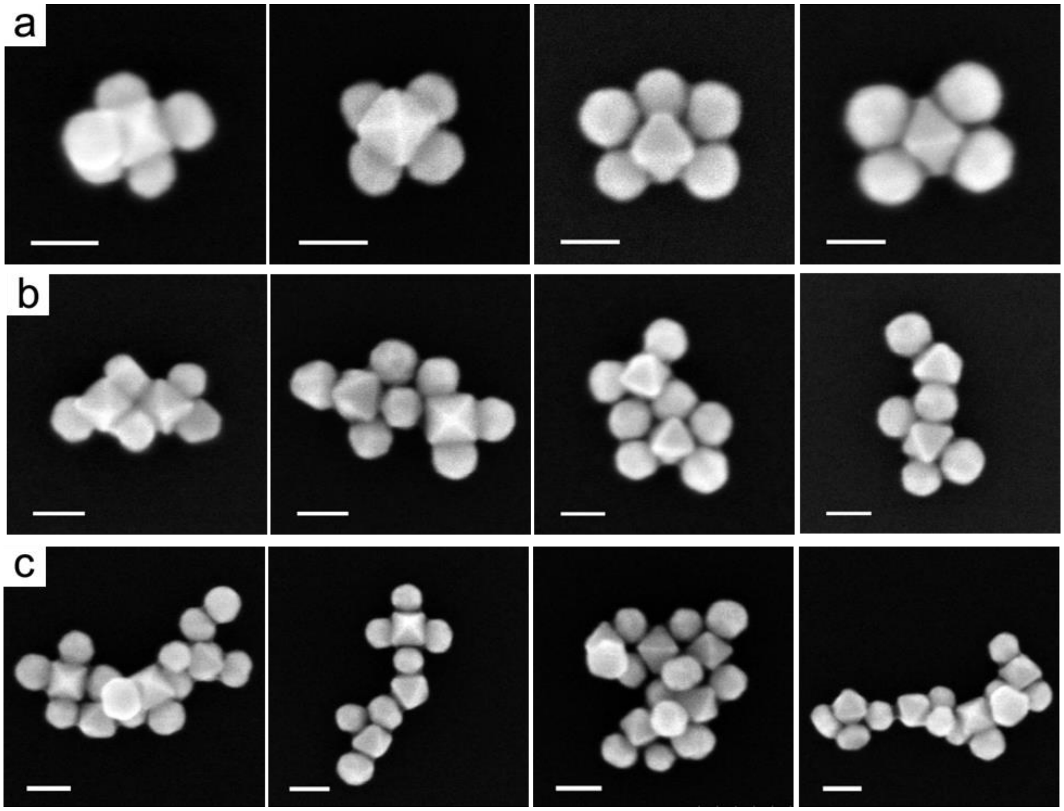

Supplementary Figure 8. Octahedron-encoded assemblies of clusters. Representative SEM images illustrating evolution stages (from **row a, b,** to **c**) of the clusters assembled from the 46 nm SNPs/46 nm OCs binary-shape systems. Scale bars: 50 nm.

Supplementary Figure 9

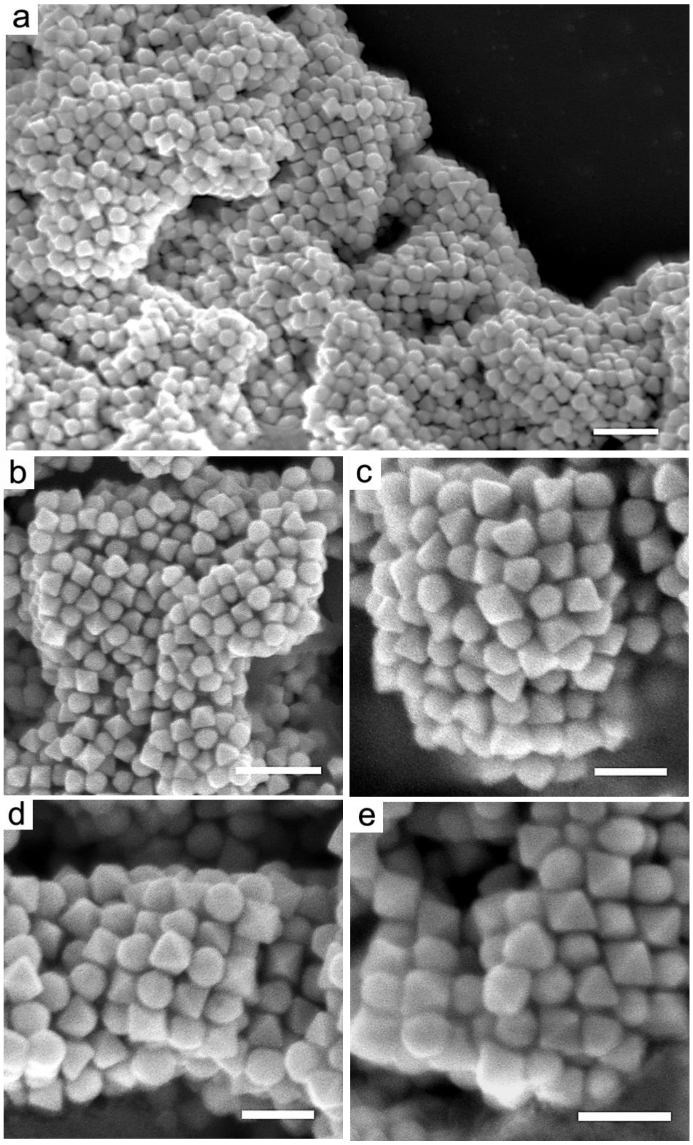

Supplementary Figure 9. Morphology of the 46 nm OCs/46 nm SNPs assemblies. Representative SEM images of the SNPs/OCs assembled superlattice revealing the coordination function of octahedrons for spheres in binary assembly systems. Scale bars represent 200 nm in **a-b** and 100 nm in **c-e**, respectively. Note that, in the formed CsCl-type lattices, one octahedron is oriented tip-to-tip with its nearest neighbor octahedrons and thus slips easily from their contacting points when lattices shrink, which might make it difficult to preserve their ordered morphologies in the dry samples.

Supplementary Table 1. DNA sequences used in the study

DNA	Sequence
**18A**	3'-TAC TTC ACA TCC AAT TTT-C_6H_{12}-SH-5'
**18A'**	3'-GAA GTA ACA TCC AAT TTT-C_6H_{12}-SH-5'
**8-base duplexer strand**	3'-ATTGGATG-5'
**30A**	3'-TAC TTC CAA TCC AAT TTT TTT TTT TTT TTT -C_6H_{12}-SH-5'
**30A'**	3'-TGG AAG TAA TCC AAT TTT TTT TTT TTT TTT -C_6H_{12}-SH-5'
**50A**	3'-TAC TTC CAA TCC AAT TCT TGT GTC GAT AGG TCG GTT GCT TTT TTT TTT TT-C_6H_{12}-SH-5'
**50A'**	3'-TGG AAG TAA TCC AAT TCT TGT GTC GAT AGG TCG GTT GCT TTT TTT TTT TT-C_6H_{12}-SH-5'
**80A**	3'-TA CTT CCA ATC CAA TTC TTG TGT CGA TAG GTC GGT TGC TTT TTT TTT TTT TTT TTT TTT TTT TTT TTT TTT TTT TTT TTT-C_6H_{12}-SH-5'
**80A'**	3'-TG GAA GTA ATC CAA TTC TTG TGT CGA TAG GTC GGT TGC TTT TTT TTT TTT TTT TTT TTT TTT TTT TTT TTT TTT TTT TTT-C_6H_{12}-SH-5'

Supplementary Note 1

A Model Based Method for Tomographic Reconstructions of Nanoparticle Assemblies

Here we used a model based tomographic method that only relies on the projected centroids of the nanoparticles and bypasses the image intensity. This method only requires 5-10 tilt images and is useful for calibrating a TEM goniometer and field/scan distortions. This method is based on measuring the geometric centers $(x^i(\theta), y^i(\theta))$ of nanoparticles in the projected images as a function of the tilt angle, θ, where the superscript *i* is the label for each individual particle. The frame of reference/rotation axis can be shifted to a specific particle *n* by a pre-subtraction procedure, i.e., $\left(x^i(\theta), y^i(\theta)\right) = \left(x^i(\theta), y^i(\theta)\right) - \left(x^n(\theta), y^n(\theta)\right)$. The three-dimensional (3D) positions of the particles at zero degree tilt in the particle *n* reference frame can be retrieved by a nonlinear least squares fitting of the below equation.

$$\begin{pmatrix} x^i(\theta) \\ y^i(\theta) \\ z^i(\theta) \end{pmatrix} = \begin{pmatrix} \cos\alpha & -\sin\alpha & 0 \\ \sin\alpha & \cos\alpha & 0 \\ 0 & 0 & 1 \end{pmatrix} \begin{pmatrix} \cos\beta & 0 & \sin\beta \\ 0 & 1 & 0 \\ -\sin\beta & 0 & \cos\beta \end{pmatrix} \begin{pmatrix} 1 & 0 & 0 \\ 0 & \cos\theta & -\sin\theta \\ 0 & \sin\theta & \cos\theta \end{pmatrix} \begin{pmatrix} x_0^i \\ y_0^i \\ z_0^i \end{pmatrix} \quad (1)$$

where, α is the image/scan rotation with respect to the projected tilt axis, and β is the offset angle between the 3D tilt axis and the projection plane (Fig. 2b). There are five unknowns— $\alpha, \beta, (x_0^i, y_0^i, z_0^i)$ —in the above equation. At each tilt, two linearly independent equations can be provided—$(x^i(\theta), y^i(\theta))$ are observables. Therefore, in principal, only three tilt images are needed to solve all the unknowns; in practice, however, additional images are needed to average out drift and scan distortions. In conjunction with the shape and size of each nanoparticle, which can be estimated from the projection images, a three dimensional model of the cluster can built.

Supplementary Note 2

Modeling of SAXS profiles from nanoparticle superlattices

In order to confirm the nanoparticle assemblies observed by SEM, we compared our experimental x-ray scattering structure factor data to the theoretical scattering for the nanoparticles on the proposed lattice. We use our recently-published scattering formalism, which simulates powder SAXS profiles for lattices of particles with arbitrary shape [1]. This formalism accounts for particle size, particle shape, and particle orientation within the unit cell. We also explicitly include disorder: particle size polydispersity, lattice disorder (Debye-Waller factor), and average grain size. The form factors, $F(\mathbf{q})$, for spheres, cubes, and octahedra, are already described in the literature; we define $P(q)$ to be the orientationally averaged form factor (which can be measured experimentally by heating the aggregates above the DNA melt temperature). The structural packing is incorporated into a lattice factor ($Z_0(q)$), assuming an isotropic distribution of grains.

$$Z_0(q) = \frac{1}{q^2} \sum_{\{hkl\}}^{m_{hkl}} \left| F(\mathbf{q}_{hkl}) \sum_{j=1}^{N_j} e^{2\pi i (x_j h + y_j k z_j l)} \right|^2 L(q - q_{hkl}) \tag{2}$$

Polydispersity is accounted for by using a Gaussian distribution of particles sizes to compute an effective $F(\mathbf{q})$ and $P(q)$. The extinction of structural scattering leads to a corresponding increase in diffuse scattering.

$$\beta(q) = \frac{|\langle F(\mathbf{q}) \rangle|^2}{\left\langle |F(\mathbf{q})|^2 \right\rangle} = \frac{|\langle F(\mathbf{q}) \rangle|^2}{P(q)} \tag{3}$$

$$G(q) = e^{-\sigma_D^2 q^2 a^2} \tag{4}$$

$$\begin{aligned} I(q) &= P(q) \left[1 + \frac{c Z_0(q)}{P(q)} G(q) - \beta(q) G(q) \right] \\ &= P(q) S(q) \end{aligned} \tag{5}$$

In our model, the structure factor $S(q)$ contains a diffuse scattering term $1-\beta(q)G(q)$ (which accounts for particle polydispersity via $\beta(q)$, and positional disorder via $G(q)$), and a structural term $cZ_0(q)G(q)/P(q)$, where c is a scaling constant. The average aggregate size for the lattices is included in the peak width via the well-known Debye-Scherrer relation [2].

The nanocubes used in this work are not perfect platonic solids; they have slightly rounded corners and edges as a result of the synthesis protocol. In order to account for this effect, the form factor for the cube-like nanoparticles was computed by using a 'superball': a mathematical equation which can be used to describe a rounded cube:

$$|x|^{2p}+|y|^{2p}+|z|^{2p}\leq 1 \tag{6}$$

The parameter p defines the shape of the object: $p=\infty$ describes an ideal cube with sharp edges, $p=1$ describes a sphere, and intermediate values ($1<p<\infty$) describe a cube whose edges have differing curvature. From SEM data, we estimate $p \sim 3$ and use this value in the scattering model. The superball form factor is computed by numerically solving the scattering integral over the volume of the superball object [3,4].

For the cube-sphere assembly, an 'alternating simple cubic' lattice was simulated, where a cubic lattice is filled with alternating spheres and cubes in all three dimensions (analogous to the atomic NaCl system). The model we use explicitly includes the anisotropic form factor, properly accounting for particle size, shape, and orientation. The overall peak positions were fitted by varying the lattice parameter, a. We used the independently measured (from SEM) size and size polydispersity values as initial guesses in our model, but allowed the nominal size to vary slightly (<10%) to account for any systematic differences between SEM and SAXS probes of size.

For the octahedron-sphere system, we assumed a BCC-like lattice where the central particle in the unit cell is distinct from its neighbors at the corner sites (analogous to the atomic CsCl crystal); we again used SEM measurements of size and polydispersity in our model. Overall the model scattering curves for both systems reproduce the essential features of the corresponding experimental in-situ $S(q)$, suggesting that the ordering observed by SEM is representative of the particle configuration in solution.

Supplementary Note 3

Calculation of interparticle distances from SAXS data

The nearest center-to-center interpartice distances (*D*cc) between SNP and ANP in the assemblies were determined by the following equations:

For NaCl lattice (the systems consisting of cubes and spheres), due to the similar form factor of cubes and spheres, the first diffraction peak q_1 arises from planes {200}, and thus $D_{cc} = 2 \cdot \pi / q_1$ and the lattice constant$a = 4 \cdot \pi / q_1$; for CsCl lattice (the systems consisting of octahedrons and spheres), due to the similar form factor of octahedrons and spheres, q_1 is diffracted from planes {110} and we have $D_{cc} = \sqrt{6} \cdot \pi / q_1$ and $a = (\sqrt{6} \cdot \frac{\pi}{q_1}) \times \frac{2}{\sqrt{3}}$.

The correlation length (ξ) was used to estimate the average grain size. According to Scherrer analysis [5], $\xi = \frac{K\lambda}{B \cos\frac{\theta}{2}} \cong \frac{2\pi K}{\delta}$, where K is a dimensionless shape factor and has a typical value of about 0.9, λ is the x-ray wavelength, B and δ are accordingly the line broadening at half the maximum intensity (FWHM) in radians and in wave vector.

Supplementary Note 4

DNA modeling for the calculation of particles surface-to-surface separation distance (D_{ss})

The surface separation distance between cube (flat surface) and sphere (curved surface), D_{ss}, was estimated from the sums of DNA shell thickness on two types of surfaces, $T_{DNA\ shell}$_Cube + $T_{DNA\ shell}$_Sphere.

The DNA shell thickness on the cube, $T_{DNA\ shell}$_Cube, was approximated using a polyelectrolyte-blob model for a flat surface [6] as $T_{\mathrm{DNA\ shell}}\text{\_Cube} = L_{\mathrm{C}}(\frac{v\sigma}{l_{\mathrm{k}}})^{1/3}$, where $L_C = N\cdot b$ is the contour length of ssDNA with $N = N_s + {}^{N_{\mathrm{D}}}/_{2}$ nucleotides of segment length b=0.65 nm (for example, DNA_18A_18A' hybridization system, the number of single-strand space-part nucleotide N_s =12 and the number of double-strand recognition-part nucleotide N_D =6, respectively). l_k ≈2 nm is the Kuhn length of ssDNA; DNA tethering density $\sigma = {}^{f}/_{6L^2}$, where f is the grafting DNA number on the nanoparticle and approximated using ~630 and L is the edge length of cube (L=46 nm). The excluded volume parameter v was estimated using Onsager's concept as $v = 1.5 \cdot l_{\mathrm{k}}^2 \cdot d_{\mathrm{eff}}$, where the ssDNA was considered as a chain of charged cylinders with a length of Kuhn length l_k and effective diameter of ssDNA $d_{\mathrm{eff}} = 1 + 2 \cdot \kappa^{-1}$. The Debye screening length $\kappa^{-1} \approx 0.3046 \cdot C_{\mathrm{a}}^{-1/2}$, where C_a = 0.2 M.

The DNA shell thickness on the sphere, $T_{DNA\ shell}$_Sphere, was approximated using Daoud-Cotton (DC) blob model for a curved surface[7] as $T_{\mathrm{DNA\ shell}}\text{\_Cube} = R[(1 + k * \frac{L_C}{R}(\frac{v\sigma}{l_{\mathrm{k}}})^{1/3})^{3/5} - 1]$. DNA tethering density $\sigma = {}^{f}/_{4\pi R^2}$, where f is approximated using ~630 and R is the radius of sphere (R=23 nm). The excluded volume parameter v was estimated using Onsager's concept as $v = 1.5 \cdot l_{\mathrm{k}}^2 \cdot d_{\mathrm{eff}}$, where the ssDNA was considered as a chain of charged cylinders with a length of Kuhn length l_k and effective diameter of ssDNA $d_{\mathrm{eff}} = 1 + 2 \cdot \kappa^{-1}$. The Debye screening length $\kappa^{-1} \approx 0.3046 \cdot C_{\mathrm{a}}^{-1/2}$, where C_a = 0.2 M. The values for D_{ss}, $T_{DNA\ shell}$_Cube, and $T_{DNA\ shell}$_Sphere are given in the table below.

The calculation of nearest surface-to-surface separation distance ($\boldsymbol{D}_{ss}$) between 46 nm sphere and 46 nm cube from DNA models and experimental SAXS data, all the values in the table are in *nm* units.

**Systems**	$\boldsymbol{T}_{\textbf{DNA shell}}$**_Cube**	$\boldsymbol{T}_{\textbf{DNA shell}}$**_Sphere**	$\boldsymbol{D}_{\textbf{ss}}$**_Model**	$\boldsymbol{D}_{\textbf{ss}}$**_SAXS**
18*R*	6.9	5.1	12	6.3
18*S*	6.9	5.1	12	13.8
30*S*	11.9	8	19.9	18.7
80*S*	34	20	54	27

Supplementary Note 5

Modeling and calculation of attraction potential energy between cube and sphere

The free energy of our DNA-NP systems mainly includes the pair attraction potential energy, ΔE_{att}, and the repulsion energy of DNA strands between same types of particles, e.g. cube and cube. Due to the small interaction area, the repulsion energy is negligible in comparison with ΔE_{att}. ΔE_{att} is dominated by the hybridization energy of DNA bridges between a sphere (radius of R) and a cube (edge length of L_{cube}), with van der Waals (vdW) interactions contributing insignificantly, and thus ΔE_{att} is proportional to the number of hybridized DNA bridges formed between the their contradictory surfaces.

A cube-sphere pair is showed as a model for calculation of attraction potential energy. The left image is a side-view and the right one is a top-view.

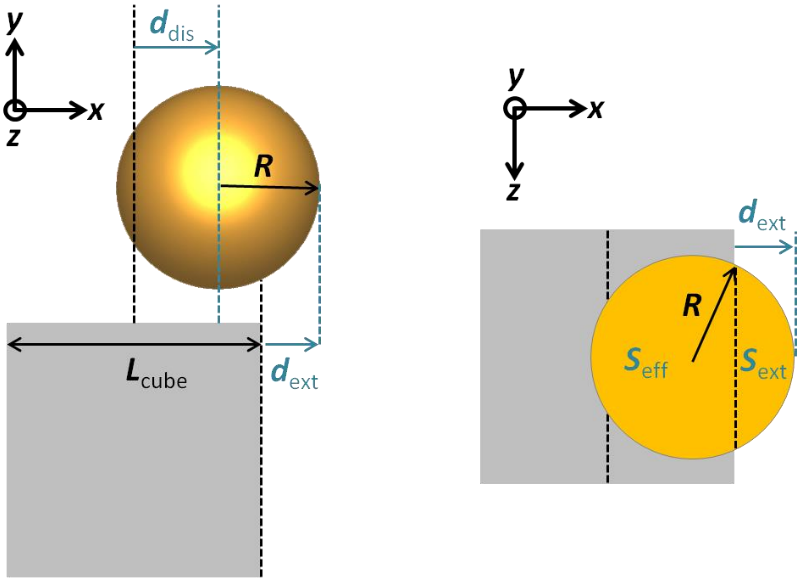

When$2R \leq L_{\text{cube}}$, the number of hybridized DNA bridges formed between sphere and cube is approximately proportional to the circle projection area of sphere on the square facet of cube, i.e., effective area, S_{eff}, which can be obtained from simple geometry considerations as:

$$S_{\text{eff}} = S_{\text{ful}} - S_{\text{ext}} \qquad (7)$$

$$S_{\text{ful}} = \pi R^2 \qquad (8)$$

$$S_{\mathrm{ext}} = R^2\left[\cos^{-1}\left(\frac{R-d_{\mathrm{ext}}}{R}\right)\right] - (R-d_{\mathrm{ext}})\sqrt{R^2-(R-d_{\mathrm{ext}})^2} \quad (9)$$

$$S_{\mathrm{eff}} = \pi R^2 + (R-d_{\mathrm{ext}})\sqrt{d_{\mathrm{ext}}(2R-d_{\mathrm{ext}})} - R^2\left[\cos^{-1}\left(\frac{R-d_{\mathrm{ext}}}{R}\right)\right] \quad (10)$$

Where S_{ful} is the surface area of the full sphere projection with radius of R; S_{ext} is the surface area of the extruding projection that is excluded from square facet of cube with a distance of d_{ext}.

As d_{dis} is the sphere displacement from the cube central axis that crosses the center of the square face, the pair attraction potential energy can be approximated as follows:

$$\Delta E_{\mathrm{att}}(R, d_{\mathrm{dis}}) \propto S_{\mathrm{eff}} = \begin{cases} \pi R^2 \quad \left(0 \le d_{\mathrm{dis}} \le \frac{L_{\mathrm{cube}}}{2} - R\right) & (11) \\ \pi R^2 + (R-d_{\mathrm{ext}})\sqrt{d_{\mathrm{ext}}(2R-d_{\mathrm{ext}})} - R^2\left[\cos^{-1}\left(\frac{R-d_{\mathrm{ext}}}{R}\right)\right], & (12) \end{cases}$$

$$d_{\mathrm{ext}} = d_{\mathrm{dis}} - \frac{L_{\mathrm{cube}}}{2} + R, \qquad \left(\frac{L_{\mathrm{cube}}}{2} - R < d_{\mathrm{dis}} \le \frac{L_{\mathrm{cube}}}{2}\right)$$

Considering compare convenience, the pair attraction potential energy can be normalized by$|\Delta E_{\mathrm{att}}(R, 0)|$, as shown in Fig.5f.

Supplementary References

1 Yager, K. G., Zhang, Y. G., Lu, F. & Gang, O. Periodic lattices of arbitrary nano-objects: modeling and applications for self-assembled systems. *Journal of Applied Crystallography* **47**, 118-129 (2014).

2 Langford, J. I. & Wilson, A. J. C. Scherrer after 60 Years - Survey and Some New Results in Determination of Crystallite Size. *Journal of Applied Crystallography* **11**, 102-113 (1978).

3 Jiao, Y., Stillinger, F. H. & Torquato, S. Optimal packings of superballs. *Physical Review E* **79** (2009).

4 Zhang, Y. G., Lu, F., van der Lelie, D. & Gang, O. Continuous Phase Transformation in Nanocube Assemblies. *Physical Review Letters* **107** (2011).

5 Patterson, A. L. The Scherrer formula for x-ray particle size determination. *Physical Review* **56**, 978-982 (1939).

6 Hariharan, R., Biver, C., Mays, J. & Russel, W. B. Ionic strength and curvature effects in flat and highly curved polyelectrolyte brushes. *Macromolecules* **31**, 7506-7513 (1998).

7 Zhang, Y. G., Lu, F., Yager, K. G., van der Lelie, D. & Gang, O. A general strategy for the DNA-mediated self-assembly of functional nanoparticles into heterogeneous systems. *Nature Nanotechnology* **8**, 865-872 (2013).
